# New algorithms for maximum disjoint paths based on tree-likeness

**DOI:** 10.1007/s10107-017-1199-3

**Published:** 2017-11-14

**Authors:** Krzysztof Fleszar, Matthias Mnich, Joachim Spoerhase

**Affiliations:** 1 Department 1: Algorithms and Complexity, Max-Planck-Institut für Informatik, Campus E1 4, 66123 Saarbrücken, Germany; 2 Department of Quantitative Economics, Maastricht University, P.O. Box 616, 6200 MD Maastricht, The Netherlands; 3 Institut für Informatik, Universität Bonn, Friedrich-Ebert-Allee 144, 53113 Bonn, Germany; 4 Lehrstuhl für Informatik I, Universität Würzburg, Am Hubland, 97074 Würzburg, Germany

**Keywords:** Disjoint paths, Approximation algorithm, Feedback vertex set, Fixed-parameter algorithm, 68-02, 68-06, 05C05, 05C21, 05C38, 05C40, 05C85, 68R10, 68W05, 68W20, 68W25, 68W40, 68Q17, 68Q25, 68Q87, 90B10, 90B18, 90C05, 90C10, 90C27, 90C35, 90C39, 90C46, 90C49, 90C59, 49L20

## Abstract

We study the classical ${\mathsf {NP}}$-hard problems of finding maximum-size subsets from given sets of *k* terminal pairs that can be routed via edge-disjoint paths (MaxEDP) or node-disjoint paths (MaxNDP) in a given graph. The approximability of MaxEDP/MaxNDP is currently not well understood; the best known lower bound is ${2^{\varOmega (\sqrt{\log n})}}$, assuming ${\mathsf {NP}\not \subseteq \mathsf {DTIME}(n^{\mathcal {O}(\log n)})}$. This constitutes a significant gap to the best known approximation upper bound of ${\mathcal {O}(\sqrt{n})}$ due to Chekuri et al. (Theory Comput 2:137–146, 2006), and closing this gap is currently one of the big open problems in approximation algorithms. In their seminal paper, Raghavan and Thompson (Combinatorica 7(4):365–374, 1987) introduce the technique of randomized rounding for LPs; their technique gives an ${\mathcal {O}(1)}$-approximation when edges (or nodes) may be used by ${\mathcal {O}\left( \log n/\log \log n\right) }$ paths. In this paper, we strengthen the fundamental results above. We provide new bounds formulated in terms of the *feedback vertex set number* *r* of a graph, which measures its vertex deletion distance to a forest. In particular, we obtain the following results:

- For MaxEDP, we give an ${\mathcal {O}(\sqrt{r} \log ({k}r))}$-approximation algorithm. Up to a logarithmic factor, our result strengthens the best known ratio ${\mathcal {O}(\sqrt{n})}$ due to Chekuri et al., as ${r\le n}$.
- Further, we show how to route ${\varOmega ({\text {OPT}}^{*})}$ pairs with congestion bounded by ${\mathcal {O}(\log (kr)/\log \log (kr))}$, strengthening the bound obtained by the classic approach of Raghavan and Thompson.
- For MaxNDP, we give an algorithm that gives the optimal answer in time ${(k+r)^{\mathcal {O}(r)}\cdot n}$. This is a substantial improvement on the run time of ${2^kr^{\mathcal {O}(r)}\cdot n}$, which can be obtained via an algorithm by Scheffler.

We complement these positive results by proving that MaxEDP is ${\mathsf {NP}}$-hard even for ${r=1}$, and MaxNDP is ${\mathsf {W}[1]}$-hard when *r* is the parameter. This shows that neither problem is fixed-parameter tractable in *r* unless ${\mathsf {FPT}= \mathsf {W}[1]}$ and that our approximability results are relevant even for very small constant values of *r*.

For MaxEDP, we give an ${\mathcal {O}(\sqrt{r} \log ({k}r))}$-approximation algorithm. Up to a logarithmic factor, our result strengthens the best known ratio ${\mathcal {O}(\sqrt{n})}$ due to Chekuri et al., as ${r\le n}$.

Further, we show how to route ${\varOmega ({\text {OPT}}^{*})}$ pairs with congestion bounded by ${\mathcal {O}(\log (kr)/\log \log (kr))}$, strengthening the bound obtained by the classic approach of Raghavan and Thompson.

For MaxNDP, we give an algorithm that gives the optimal answer in time ${(k+r)^{\mathcal {O}(r)}\cdot n}$. This is a substantial improvement on the run time of ${2^kr^{\mathcal {O}(r)}\cdot n}$, which can be obtained via an algorithm by Scheffler.

## Introduction

In this paper, we study disjoint paths routing problems. In this setting, we are given an undirected graph *G* and a collection ${\mathcal {M} = \{(s_1, t_1), \ldots , (s_k, t_k)\}}$ of vertex pairs, called *terminal pairs*, that can be thought of being source–destination pairs. The goal is to select a maximum-sized subset ${\mathcal {M}' \subseteq \mathcal {M}}$ of the pairs that can be *feasibly* *routed*, where a routing of ${\mathcal {M}'}$ is a collection ${\mathcal {P}}$ of paths such that, for each pair ${(s_i, t_i) \in \mathcal {M}'}$, there is a path in ${\mathcal {P}}$ connecting ${s_i}$ to ${t_i}$. In the Maximum Edge Disjoint Paths (MaxEDP) problem, a routing ${\mathcal {P}}$ is feasible if its paths are pairwise edge-disjoint, and in the Maximum Node Disjoint Paths (MaxNDP) problem, a routing ${\mathcal {P}}$ is feasible if its paths are pairwise node-disjoint. Throughout this paper, a *solution* to MaxEDP or MaxNDP is a feasible routing ${\mathcal {P}}$ of a subset ${\mathcal {M}' \subseteq \mathcal {M}}$.

Disjoint paths problems are fundamental problems with a long history and significant connections to optimization and structural graph theory. The decision versions EDP of MaxEDP and NDP of MaxNDP ask whether all of the pairs can be routed. When the number of pairs is part of the input, EDP and NDP are ${\mathsf {NP}}$-complete [22, 29]. In undirected graphs, MaxEDP and MaxNDP are solvable in polynomial time when the number of pairs is a fixed constant; this is a very deep result of Robertson and Seymour [42] that builds on several fundamental results in structural graph theory from their graph minors project.

In this paper, we consider the optimization problems MaxEDP and MaxNDP when the number of pairs is part of the input. In this setting, the best approximation ratio for MaxEDP is achieved by an ${\mathcal {O}(\sqrt{n})}$-approximation algorithm [11, 35], that is, by an algorithm that routes ${{\varOmega }({\text {OPT}}/\sqrt{n})}$ pairs, where ${{\text {OPT}}}$ is the number of pairs in an optimum routing and *n* is the number of nodes. However, the best known lower bound for undirected graphs is only ${2^{\varOmega (\sqrt{\log n})}}$, assuming ${\mathsf {NP}\not \subseteq \mathsf {DTIME}(n^{\mathcal {O}(\log n)})}$ [19]. Bridging this gap is a fundamental open problem that seems quite challenging.

Most of the results for routing on disjoint paths use a natural multi-commodity flow relaxation as a starting point. A well-known integrality gap instance due to Garg et al. [26] shows that this relaxation has an integrality gap of ${\varOmega (\sqrt{n})}$, and this is the main obstacle for improving the ${\mathcal {O}(\sqrt{n})}$-approximation ratio in general graphs. This led Chekuri et al. [15] to study the approximability of MaxEDP with respect to the *treewidth* of the underlying graph. In particular, they pose the following conjecture:

### Conjecture 1

[12] The integrality gap of the standard multi-commodity flow relaxation for MaxEDP is ${\varTheta (w)}$, where *w* is the treewidth of the graph.

Recently, Ene et al. [21] showed that MaxEDP admits an ${\mathcal {O}(w^3)}$-approximation algorithm on graphs of treewidth at most *w*. Theirs is the best known approximation ratio in terms of *w*, improving on an earlier ${\mathcal {O}(w\cdot 3^w)}$-approximation algorithm due to Chekuri et al. [15]. This shows that the problem seems more amenable on “tree-like” graphs.

However, for ${w=\omega (n^{1/6})}$, the bound is weaker than the bound of ${\mathcal {O}(\sqrt{n})}$. In fact, EDP remains ${\mathsf {NP}}$-hard even for graphs of *constant* treewidth, namely treewidth ${w = 2}$ [39]. This further rules out the existence of a fixed-parameter algorithm for MaxEDP parameterized by treewidth, assuming ${\mathsf {P}\not =\mathsf {NP}}$. Therefore, to obtain fixed-parameter tractability results as well as better approximation guarantees, one needs to resort to parameters stronger than treewidth.

Another route to bridge the large gap between approximation lower and upper bounds for MaxEDP is to allow the paths to have *congestion c*: that is, instead of requiring the routed paths to be pairwise disjoint, at most *c* paths can use an edge. We can also think of this problem that each edge has a *capacity* *c*; thus, on unit-capacity graphs we ask for solutions without congestion. In their groundbreaking work, Raghavan and Thompson [40] introduced the technique of randomized rounding of LPs to obtain polynomial-time approximation algorithms for combinatorial problems. Their approach allows to route ${\varOmega ({\text {OPT}}^{*})}$ pairs of paths with congestion ${\mathcal {O}\left( \log {n}/\log \log {n}\right) }$, where ${{\text {OPT}}^{*}}$ denotes the value of an optimum solution to the multi-commodity flow relaxation. This extensive line of research [2, 17, 31] has culminated in a ${\log ^{\mathcal {O}(1)} k}$-approximation algorithm with congestion 2 for MaxEDP [20]. A slightly weaker result also holds for MaxNDP [10].

### Motivation and contribution

The goal of this work is to study disjoint paths problems under another natural measure for how “far” a graph is from being a tree. In particular, we propose to examine MaxEDP and MaxNDP under the *feedback vertex set number*. It denotes the smallest size *r* of a *feedback vertex set* of a graph *G*, which is a subset *R* of nodes for which ${G - R}$ is a forest. Note that the treewidth of *G* is at most ${r+1}$. Therefore, given the ${\mathsf {NP}}$-hardness of EDP for treewidth ${w = 2}$ and the current gap between the best known upper bound ${\mathcal {O}(w^3)}$ and the linear upper bound suggested by Conjecture 1, it is interesting to study the stronger restriction of bounding the feedback vertex set number *r* of the input graph. Our approach is further motivated by the fact that MaxEDP is efficiently solvable on trees by means of the algorithm of Garg et al. [26]. Similarly, MaxNDP is easy on trees (see Theorem 3). Throughout this work, the parameter *r* will denote the feedback vertex set number of a graph.

Our main insight is that one can in fact obtain bounds in terms of *r* that either strengthen the best known bounds or are almost tight (see Table 1). It therefore seems that the parameter *r* correlates quite well with the “difficulty” of disjoint paths problems.

Our first result allows the paths to have small congestion: in this setting, we strengthen the result, obtained by the classic randomized LP-rounding approach of Raghavan and Thompson [40], that one can always route ${\varOmega ({\text {OPT}}^{*})}$ pairs with congestion ${\mathcal {O}\left( \log {n}/\log \log {n}\right) }$ with constant probability.

#### Theorem 1

There is a polynomial-time algorithm for MaxEDP that produces—with constant probability—a routing of ${\varOmega ({\text {OPT}}^{*})}$ paths with congestion ${\mathcal {O}\left( \log ({k}r)/\log \log ({k}r)\right) }$ where ${{\text {OPT}}^{*}}$ is the value of an optimum solution to the multi-commodity flow relaxation, *k* is the number of terminal pairs and *r* is the feedback vertex set number.

In other words, we show that there is an ${\mathcal {O}(1)}$-approximation algorithm for MaxEDP with congestion ${\mathcal {O}\left( \log ({k}r)/\log \log ({k}r)\right) }$.

Our second main result builds upon Theorem 1 and uses it as a subroutine. We show how to use a routing for MaxEDP with low congestion to obtain a polynomial-time approximation algorithm for MaxEDP *without congestion* that performs well in terms of *r*.

#### Theorem 2

There is a polynomial-time algorithm for MaxEDP that produces—with constant probability—a routing of ${{\text {OPT}}^{*}\!\!/ \mathcal {O}(\sqrt{r}\log (kr))}$ paths with no congestion where ${{\text {OPT}}^{*}}$ is the value of an optimum solution to the multi-commodity flow relaxation, *k* is the number of terminal pairs and *r* is the feedback vertex set number.

In particular, our algorithm strengthens the best known approximation algorithm for MaxEDP on general graphs [11] as always ${r\le n}$, and indeed it matches that algorithm’s performance up to a logarithmic factor. Substantially improving upon our bounds would also improve the current state of the art of MaxEDP. Conversely, the result implies that it suffices to study graphs with close to linear feedback vertex set number in order to improve the currently best upper bound of ${\mathcal {O}(\sqrt{n})}$ on the approximation ratio [11].

Our algorithmic approaches harness the forest structure of ${G - R}$ for any feedback vertex set *R*. However, the technical challenge comes from the fact that the edge set running between ${G - R}$ and *R* is unrestricted. Therefore, the “interaction” between *R* and ${G - R}$ is non-trivial, and flow paths may run between the two parts in an arbitrary manner and multiple times. In fact, we show that MaxEDP is already ${\mathsf {NP}}$-hard if *R* consists of a *single node* (Theorem 5); this contrasts the efficient solvability on forests [26].

In order to overcome the technical hurdles, we propose several new concepts, which we believe could be of interest in future studies of disjoint paths or routing problems.

In the randomized rounding approach of Raghavan and Thompson [40], it is shown that the probability that the congestion on any fixed edge is larger than ${c\log n/\log \log n}$ for some constant *c* is at most ${1/n^{\mathcal {O}(1)}}$. Combining this with the fact that there are at most ${n^2}$ edges, yields that every edge has bounded congestion with high probability. The number of edges in the graph may, however, be unbounded in terms of *r* and *k*. Hence, in order to prove Theorem 1, we propose a non-trivial *preprocessing step* of the optimum LP solution that is applied prior to the randomized rounding. In this step, we aggregate the flow paths by a careful rerouting so that the flow “concentrates” in ${\mathcal {O}(kr^2)}$ nodes (so-called *hot spots*) in the sense that if all edges incident on hot spots have low congestion, then so have all edges in the graph. Unfortunately, for any such hot spot the number of incident edges carrying flow may still be unbounded in terms of *k* and *r*. We are, however, able to give a refined probabilistic analysis that suitably relates the probability of exceeding the congestion bound to the amount of flow on the respective edge. Since the total amount of flow traversing any given hot spot is at most *k*, the probability that there is an edge incident on this hot spot that violates the congestion bound is inverse polynomial in *r* and *k*.

The known ${\mathcal {O}(\sqrt{n})}$-approximation algorithm for MaxEDP by Chekuri et al. [11] employs a clever LP-rounding approach. If there are many long flow paths in the LP solution, then there must be a single node carrying a significant fraction of the total flow and a good fraction of this flow can be realized by integral paths by solving a single-source flow problem. If the LP solution contains many short flow paths, then greedily routing these short paths yields the bound. Essentially, this follows from the fact that routing a short path blocks only a small amount of flow. In order to prove Theorem 2, we also distinguish two cases. We are interested, however, in the number of nodes in *R* that a flow path is visiting rather than in its length. In the first case, there are many paths, each of which is visiting a large number of nodes in *R*. Here, we reduce to a single-source flow problem in a similar way to the approach of Chekuri et al. The second case where a majority of the flow paths visit only a few nodes in *R* turns out to be more challenging, since any such path may still visit an unbounded number of edges in terms of *k* and *r*. We use two main ingredients to overcome these difficulties. First, we apply our Theorem 1 as a building block to obtain a solution with logarithmic congestion while losing only a constant factor in the approximation ratio. Secondly, we introduce the concept of *irreducible routings with low congestion* which allows us to exploit the structural properties of the graph and the congestion property to identify a sufficiently large number of flow paths blocking only a small amount of flow.

Note that the natural greedy approach of always routing the shortest conflict-free path gives only an approximation ratio of ${\mathcal {O}(\sqrt{m})}$ for MaxEDP, where *m* is the number of edges. We believe that it is non-trivial to obtain our bounds via a more direct or purely combinatorial approach.

Our third result is a fixed-parameter algorithm for MaxNDP in ${k + r}$.

#### Theorem 3

MaxNDP can be solved in time ${(k+r)^{\mathcal {O}(r)}\cdot n}$ on graphs with *k* terminal pairs, feedback vertex set number *r*, and *n* vertices. When a minimum feedback vertex set is given, it can be even solved in time ${(8k+8r)^{2r+3}\cdot \mathcal {O}(n)}$.

This run time is polynomial for constant *r*. We also note that, for small *r*, our algorithm is asymptotically significantly faster than the fastest known algorithm for NDP, by Kawarabayashi and Wollan [30], which requires time at least *quadruple-exponential* in *k* [1]. Namely, if *r* is asymptotically less than triple-exponential in *k*, our algorithm is asymptotically faster than theirs. We achieve this result by the idea of so-called *essential pairs* and *realizations*, which characterizes the “interaction” between the feedback vertex set *R* and the paths in an optimum solution. Note that in our algorithm of Theorem 3 the parameter *k* does not appear in the exponent of the run time at all. Hence, whenever ${r =o(k/\log k)}$, our algorithm is asymptotically faster than reducing MaxNDP to NDP by guessing the subset of pairs to be routed (at an expense of ${2^k}$ in the run time) and using Scheffler’s [43] algorithm for NDP with run time ${2^{\mathcal {O}(r\log r)}\cdot n}$; for $r=\varOmega (k/\log k)$, our algorithm is asymptotically not slower.

Once a fixed-parameter algorithm for a problem has been obtained, the existence of a polynomial-size kernel comes up. Here we note that MaxNDP does not admit a polynomial kernel for the combined parameter ${k+r}$, unless ${\mathsf {NP}\subseteq \mathsf {coNP}/\mathrm {poly}}$ [7].

Another natural question is whether the run time ${f(k,r)\cdot n}$ in Theorem 3 can be improved to ${f(r)\cdot n^{\mathcal {O}(1)}}$. We answer this question in the negative, ruling out the existence of a fixed-parameter algorithm for MaxNDP parameterized by *r* (assuming ${\mathsf {FPT}\not =\mathsf {W}[1]}$):

#### Theorem 4

MaxNDP in unit-capacity graphs is ${\mathsf {W}[1]}$-hard parameterized by feedback vertex set number.

This contrasts the known result that NDP is fixed-parameter tractable in feedback vertex set number  [43]—which further stresses the relevance of understanding this parameter.

For MaxEDP, we prove that the situation is, in a sense, even worse:

#### Theorem 5

MaxEDP is ${\mathsf {NP}}$-hard for unit-capacity graphs with feedback vertex set number ${r = 1}$ and EDP is ${\mathsf {NP}}$-hard for unit-capacity graphs with feedback vertex set number ${r=2}$.

This theorem also shows that our algorithms are relevant for small values of *r*, and that they nicely complement the ${\mathsf {NP}}$-hardness for MaxEDP in capacitated trees [26].

Our results are summarized in Table 1.

**Table 1:** Summary of results obtained in this paper

	EDP	MaxEDP	NDP	MaxNDP
$r = 0$	Poly [26]	Poly [26]	Poly [43]	Poly (Thm. 3)
$r = 1$	*Open*	$\mathsf {NP}$-hard (Thm. 5)	Poly [43]	Poly (Thm. 3)
Const. $r \ge 2$	${\mathsf {NP}}$-hard (Thm. 5)	$\mathsf {NP}$-hard (Thm. 5)	Poly [43]	Poly (Thm. 3)
Param. *r*	Para-$\mathsf {NP}$-hard (Thm. 5)		$\mathsf {FPT}$ [43]	$\mathsf {W}[1]$-hard (Thm. 4)
	$\mathcal {O}(\sqrt{r}\log ({kr}))$-approx (Thm. 2)		Exact ${(k+r)^{\mathcal {O}(r)} n}$ (Thm. 3)	
	$\mathcal {O}(1)$-approx. w. cg. $\mathcal {O}\left( \frac{\log ({k}r)}{\log \log ({k}r)}\right)$ (Thm. 1)			

*Related work* Our study of the parameter feedback vertex set number is in line with the general attempt to obtain bounds for MaxEDP (or related problems) that are independent of the input size. Besides the above-mentioned works that provide bounds in terms of the treewidth of the input graph, Günlük [27] and Chekuri et al. [16] give bounds on the *flow-cut gap* for the closely related integer multi-commodity flow problem; their bounds are logarithmic with respect to the *vertex cover number* of a graph. This improved upon earlier bounds of ${\mathcal {O}(\log n)}$ [36] and ${\mathcal {O}(\log k)}$ [4, 37]. As every vertex cover is in particular a feedback vertex set of a graph, our results for disjoint path problems address a generalization of graphs with bounded vertex cover number. Bodlaender et al. [7] showed that NDP does not admit a polynomial kernel parameterized by vertex cover number *and* the number *k* of terminal pairs, unless ${\mathsf {NP}\subseteq \mathsf {coNP}/\mathrm {poly}}$; therefore, NDP is unlikely to admit a polynomial kernel in ${k + r}$ either. Ene et al. [21] showed that MaxNDP is ${\mathsf {W}[1]}$-hard parameterized by tree-depth, which is another restriction of treewidth that is incomparable to feedback vertex set number.

The basic gap in understanding the approximability of MaxEDP has led to several improved results for special graph classes, and also our results can be seen in this light. For example, polylogarithmic approximation algorithms are known for graphs whose global minimum cut value is ${\varOmega (\log ^5 n)}$ [41], for bounded-degree expanders [8, 9, 25, 32, 36], and for Eulerian planar or 4-connected planar graphs [31]. Constant factor approximation algorithms are known for capacitated trees [13, 26], grids and grid-like graphs [3, 5, 33, 34]. For planar graphs, there is a constant-factor approximation algorithm with congestion 2 [44]. Very recently, Chuzhoy et al. [18] gave a ${\tilde{\mathcal {O}}(n^{9/19})}$-approximation algorithm for MaxNDP on *planar* graphs. However, improving the ${\mathcal {O}(\sqrt{n})}$-approximation algorithm for MaxEDP remains elusive even for planar graphs.

## Preliminaries

We use standard graph theoretic notation. For a graph *G*, let *V*(*G*) denote its vertex set and *E*(*G*) its edge set. The *length* of a path is the number of its edges. A *feedback vertex set* of a graph *G* is a set ${R\subseteq V(G)}$ such that ${G - R}$ is a forest. A *minor* of a graph *G* is a graph *H* that is obtained by successively contracting edges from a subgraph of *G* (and deleting any occurring loops). A class ${\mathcal {G}}$ of graphs is *minor-closed* if for any graph in ${\mathcal {G}}$ also all its minors belong to ${\mathcal {G}}$.

For an instance ${(G,\mathcal {M})}$ of MaxEDP/MaxNDP, we refer to the vertices participating in the pairs ${\mathcal {M}}$ as *terminals*. It is convenient to assume that ${\mathcal {M}}$ forms a matching on the terminals; this can be ensured by making several copies of the terminals and attaching them as leaves. Hence, we can also assume that all terminals are leaves.

*Multi-commodity flow relaxation* We use the following standard multi-commodity flow relaxation for MaxEDP that we will call MaxEDP LP (there is an analogous relaxation for MaxNDP). We use ${\mathcal {P}(u, v)}$ to denote the set of all paths in *G* from *u* to *v*, for each pair (*u*, *v*) of nodes. Since the pairs in ${\mathcal {M}}$ form a matching, the sets in ${\{\mathcal {P}(s_i,t_i) \mid (s_i,t_i)\in \mathcal {M}\}}$ are pairwise disjoint. Let ${\mathcal {P} = \bigcup _{i = 1}^k \mathcal {P}(s_i, t_i)}$. The LP has a variable *f*(*P*) for each path ${P \in \mathcal {P}}$ representing the amount of flow on *P*. For each pair ${(s_i, t_i) \in \mathcal {M}}$, the LP has a variable ${x_i}$ denoting the total amount of flow routed for the pair (in the corresponding integer program, $x_i$ denotes whether the pair is routed or not). The LP imposes the constraint that there is a flow from ${s_i}$ to ${t_i}$ of value ${x_i}$. Additionally, the LP has constraints that ensure that the total amount of flow on paths using a given edge (respectively node for MaxNDP) is at most 1.

$$\begin{aligned} \text {maximize } \quad \sum _{i = 1}^k x_i \\ \text {subject to } \sum _{P \in \mathcal {P}(s_i, t_i)} f(P)= & {} x_i \le 1 \quad \text {for each } i=1,\ldots ,k;\\ \sum _{P \in \mathcal {P}:\; e \in P} f(P)\le & {} 1 \quad \text {for each } e \in E(G);\\ f(P)\ge & {} 0 \quad \text {for each } P \in \mathcal {P}. \end{aligned}$$

It is well-known that the relaxation MaxEDP LP can be solved in polynomial time, since there is an efficient separation oracle for the dual LP (alternatively, one can write a compact relaxation). We use ${(f, \mathbf {x})}$ to denote a feasible solution to MaxEDP LP for an instance ${(G, \mathcal {M})}$ of MaxEDP.

**Fig. 1:**
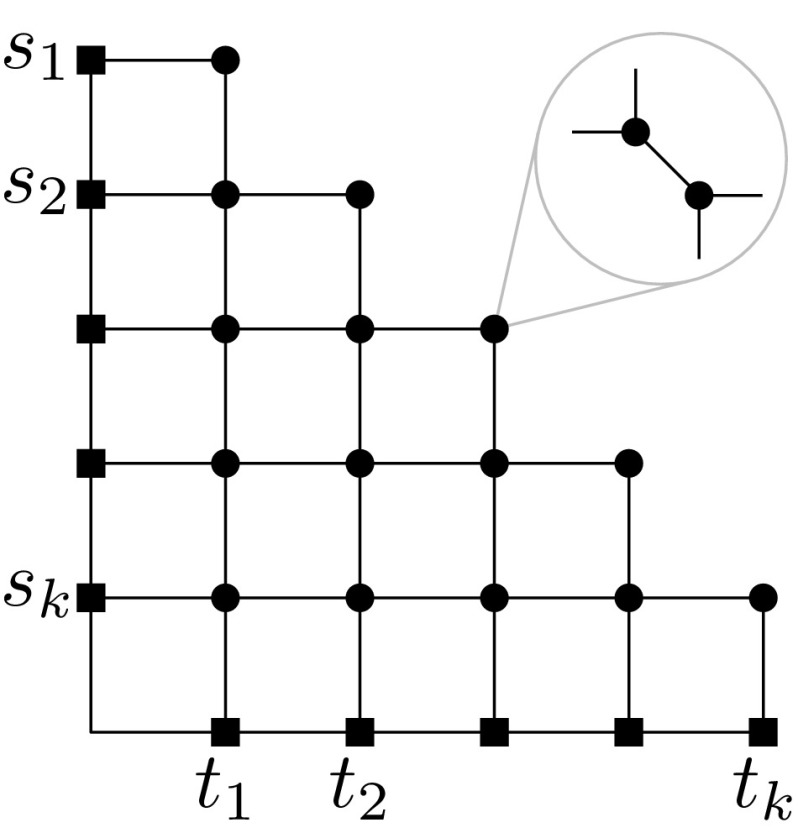
An instance with an integrality gap of ${\varOmega (\sqrt{n})}$ for MaxEDP [26]: Any integral routing routes at most one pair, whereas a fractional multi-commodity flow can send 1 / 2 unit of flow for each pair ${(s_i, t_i)}$ along the canonical path from ${s_i}$ to ${t_i}$ in the grid

As noted in the introduction, MaxEDP LP has an integrality gap of ${\varOmega (\sqrt{n})}$ as shown by Garg et al. [26]. The integrality instance on an ${n \times n}$ grid (of treewidth ${\varTheta (\sqrt{n})}$) exploits a topological obstruction in the plane that prevents a large integral routing; see Fig. 1.

We will use the following result by Chekuri et al. [11, Sect. 3.1]; see also Proposition 3.3 of Chekuri et al. [14].

### Proposition 1

(Chekuri et al. [11]) Let ${(f,\mathbf {x})}$ be a fractional solution to the LP relaxation of a MaxEDP instance ${(G,\mathcal {M})}$. If some node *v* is contained in all flow paths of *f*, then we can find an integral routing of size at least ${\sum _{i}x_i/12}$ in polynomial time.

As a corollary of Theorem 2, we immediately obtain the following proposition about the integrality gap of MaxEDP LP.

### Corollary 1

The integrality gap of the multi-commodity flow relaxation for MaxEDP with *k* terminal pairs is ${\mathcal {O}(\sqrt{r}\log (kr))}$ for graphs with feedback vertex set number *r*.

Let *f* be a multi-commodity flow assigning to each path $P\in \mathcal {P}$ a non-negative flow value *f*(*P*). The flow *f* is said to have *congestion c* if it satisfies a modification of MaxEDP LP where we replace, for each edge ${e \in E(G)}$, the constraint ${\sum _{P \in \mathcal {P}:\; e \in P} f(P) \le 1}$ with ${\sum _{P \in \mathcal {P}:\; e \in P} f(P) \le c}$. In the particular case where *f* is integral we also speak of a *routing f with congestion c*.

## Bi-criteria approximation for MaxEDP with low congestion

We present a randomized rounding algorithm that will lead to the proof of Theorem 1. First we will modify a fractional solution to the multi-commodity flow relaxation and then run a randomized rounding procedure.

### Algorithm

Consider an instance ${(G,\mathcal {M})}$ of MaxEDP. Let *k* denote the number of terminal pairs in $\mathcal {M}$, and let *R* be a feedback vertex set of *G* that we construct by taking the union of the terminals in $\mathcal {M}$ and any 2-approximate minimum feedback vertex set; note that such an approximation can be obtained in polynomial time [6]. Thus, ${|R|\le 2r+2k}$.

First, solve the corresponding MaxEDP LP. We obtain an optimal extreme point solution ${(f,\mathbf {x})}$. For each ${(s_i, t_i)\in \mathcal {M}}$, this gives us a set $\mathcal {P}'(s_i, t_i)$ of positive weighted paths that satisfy the LP constraints. Formally,

$$\begin{aligned} \mathcal {P}'(s_i, t_i) = \{P \in \mathcal {P}(s_i, t_i) \mid ~f(P)>0\}. \end{aligned}$$

Since we have an extreme point solution, the number of tight constraints is not smaller than the number of variables. Hence, given the numbers of constraints and variables, the number of constraints that are not tight is polynomially bounded in the input size. Consequently, the same bound holds for the cardinality of the set ${\mathcal {P}' = \bigcup _{i = 1}^k \mathcal {P}'(s_i, t_i)}$. In what follows, we will modify ${\mathcal {P}'}$ and then select an (unweighted) subset ${\mathcal {P}_{\text {Sol}}'}$ of ${\mathcal {P}'}$ that will form our integral solution.

Each ${P\in \mathcal {P'}}$ has the form ${(r_1,\dots ,r_2,\dots ,r_\ell )}$ where ${r_1,\dots ,r_\ell }$ are the nodes in *R* that are traversed by *P* in this order. For every *j* with ${1\le j \le \ell -1}$, we call the path ${(r_j,\dots ,r_{j+1})}$ a *subpath of* *P*. For every subpath ${P'}$ of *P*, we set ${f(P')=f(P)}$. Let ${\mathcal {S}}$ be the multi-set of all subpaths of all paths in ${\mathcal {P}'}$. Let ${F = G-R}$ be the forest obtained by removing *R*.

We now modify some paths in ${\mathcal {P}'}$, one by one, and at the same time, we incrementally construct a subset ${H_{\text {Alg}}\subseteq V(F)}$ in several steps. We will refer to the nodes in ${H_{\text {Alg}}}$ as *hot spots*. When the construction of ${H_{\text {Alg}}}$ is complete, every subpath in ${\mathcal {S}}$ will contain at least one hot spot, that is, a node in ${H_{\text {Alg}}}$.

Initially, let ${H_{\text {Alg}}=\emptyset }$. Consider any tree *T* in *F* and fix any of its nodes as a root. Then let ${\mathcal {S}_T}$ be the multi-set of all subpaths in ${\mathcal {S}}$ that, excluding the endpoints, are contained in *T*. For each subpath ${P\in \mathcal {S}_T}$, define its *highest node* *h*(*P*) as the node on *P* closest to the root. Note that ${P\cap T}$ equals ${P\cap F}$ and that ${P\cap T}$ is a path. Now, pick a subpath ${P\in \mathcal {S}_T}$ that does not contain any node in ${H_{\text {Alg}}}$ and whose highest node *h*(*P*) is *farthest away* from the root. Consider the multi-set ${\mathcal {S}[P]}$ of all subpaths in ${\mathcal {S}_T}$ that are identical to *P* (but may be subpaths of different flow paths in ${\mathcal {P}'}$). Note that the weight ${f(\mathcal {S}[P])}$ of ${\mathcal {S}[P]}$ defined as ${\sum _{P\in \mathcal {S}[P]} f(P)}$ is at most 1 by the constraints of the LP. Let ${u,v\in R}$ be the endpoints of *P*. We define ${\mathcal {S}_{uv}}$ as the set of all subpaths in ${\mathcal {S}{\setminus } \mathcal {S}[P]}$ that have *u* and *v* as their endpoints and that do not contain any node in ${H_{\text {Alg}}}$.

**Fig. 2:**
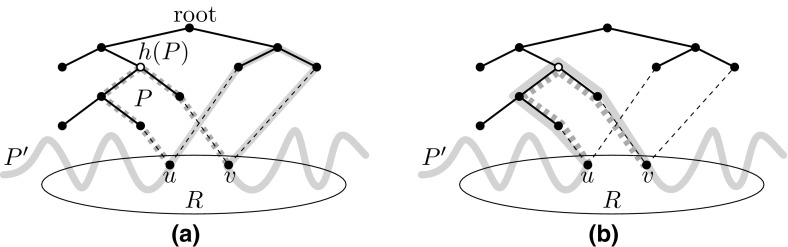
Example of the flow aggregation step: **a** A subpath *P* (highlighted in dashed gray) enters a tree (solid black edges) where *h*(*P*) (white node) is its closest node to the root. A path $P'$ (highlighted in solid gray) contains a different subpath with the same endpoints ${u,v\in R}$ as *P*. **b** We reroute $P'$ by replacing its subpath between *u* and *v* with a copy of *P*

Intuitively speaking, we now aggregate flow on *P* by rerouting as much flow as possible from ${\mathcal {S}_{uv}}$ to *P*. To this end, we repeatedly perform the following operation as long as ${f(\mathcal {S}[P])<1}$ and ${\mathcal {S}_{uv}\not =\emptyset }$. We pick a path ${P'}$ in ${\mathcal {S}}$ that contains a subpath in ${\mathcal {S}_{uv}}$; see Fig. 2. We reroute flow from ${P'}$ by creating a new path ${P''}$ that arises from ${P'}$ by replacing its subpath between *u* and *v* with a new path identical to *P*, and assign it the weight ${f(P'')}$ equal to ${\min \{f(P'),1-f(\mathcal {S}[P])\}}$. Then we set the weight of (the original path) $P'$ to ${\max \{0, f(P')+f(\mathcal {S}[P])-1\}}$. We update the sets ${\mathcal {P'}}$, ${\mathcal {P}'(s_i,t_i)}$, ${\mathcal {S}}$, ${\mathcal {S}_T}$, $\mathcal {S}[P]$ and ${\mathcal {S}_{uv}}$ accordingly.

As soon as ${f(\mathcal {S}[P])=1}$ or ${\mathcal {S}_{uv}=\emptyset }$, we mark *h*(*P*) as a hot spot and add it to ${H_{\text {Alg}}}$. Then, we proceed with the next ${P\in \mathcal {S}_T}$ that does not contain a hot spot and whose highest node *h*(*P*) is farthest away from the root. If no such *P* is left, we consider the next tree *T* in *F*.

At the end, we create our solution ${\mathcal {P}_{\text {Sol}}'}$ by randomized rounding: We route every terminal pair ${(s_i, t_i)}$ with probability ${x_i}$. In case ${(s_i,t_i)}$ is routed, we randomly select a path from ${\mathcal {P}'(s_i, t_i)}$ and add it to ${\mathcal {P}_{\text {Sol}}'}$ where the probability that the path *P* is taken is ${f(P)/x_i}$.

### Analysis

First, observe that ${\mathbf {x}}$ did not change during our modifications of the paths, as the total flow between any terminal pair did not change. Thus, the expected number of pairs routed in our solution ${\mathcal {P}_{\text {Sol}}'}$ is ${\sum _{i=1}^k x_i\ge {\text {OPT}}^{*}}$. Using the Chernoff bound, the probability that we route less than ${{\text {OPT}}^{*}\!\!/2}$ pairs is at most ${e^{-1/8{\text {OPT}}^{*}\!\!}<1/2}$, assuming ${{\text {OPT}}^{*}>8}$.

In the above algorithm, we guarantee that when we aggregate flow on a path *P*, then the total amount of all flow paths containing *P* as a subpath has increased to at most 1. Nevertheless, the flow *f* may have congestion greater than 1 after this modification. This is because *P* may intersect flow paths that contain only a proper subset of the edges of *P*. For instance, consider the situation where we increase ${f(\mathcal {S}[P'])}$ for a subpath $P'$ that initially contained a tight edge *e* (that is, an edge *e* with ${\sum _{P \in \mathcal {P}:\; e \in P} f(P) = 1}$). After increasing ${f(\mathcal {S}[P'])}$, the total amount of flow paths going through *e* is greater than 1. However, the congestion of the modified flow *f* is always at most 2 as shown by the following lemma.

#### Lemma 1

The congestion of the flow *f* is at most 2.

#### Proof

In our algorithm, we increase the flow only along flow subpaths that are pairwise edge-disjoint. To see this, consider two distinct flow subpaths *P* and ${P'}$ on which we increase the flow. If there were an edge *e* lying on *P* and ${P'}$, then both subpaths traverse the same tree in the forest *F*. Assume, without loss of generality, that *P* was considered before ${P'}$ by the algorithm. Then the path from *e* to the root would first visit *h*(*P*) and then ${h(P')}$. Hence, *h*(*P*) would be an internal node of ${P'}$. This yields a contradiction, as *h*(*P*) was already marked as a hot spot when ${P'}$ was considered. This shows that we increased the flow along any edge by at most one unit. Hence, *f* has congestion at most 2. $\square$

We now bound the congestion of the integral solution obtained by randomized rounding. In the algorithm, we constructed a set ${H_{\text {Alg}}}$ of hot spots. As a part of the analysis, we will now extend this set to a set ${H}$ as follows. Initially, ${H=H_{\text {Alg}}}$. We build a sub-forest ${F'}$ of *F* consisting of all edges of *F* that lie on a path connecting two hot spots. Then we add to ${H}$ all nodes that have degree at least 3 in ${F'}$. Since the number of nodes of degree 3 in any forest is at most its number of leaves and since every leaf of ${F'}$ is a hot spot, it follows that this can at most double the size of ${H}$ to ${2|H_{\text {Alg}}|}$. Finally, we add all nodes of the feedback vertex set *R* to ${H}$ and mark all nodes in ${H}$ as hot spots.

#### Lemma 2

The number ${|H|}$ of hot spots is at most ${2 k|R|^2 + |R|}$.

#### Proof

To this end, fix two nodes ${u,v\in R}$ and consider the set of flow subpaths with endpoints *u* and *v* for which we added their hot spots to ${H_{\text {Alg}}}$. Due to the aggregation of flows in our algorithm, all except possibly one of the subpaths are saturated, that is, they carry precisely one unit of flow. Since no two of these subpaths are contained in a same flow path of *f* and since the flow value of *f* is bounded from above by *k*, we added at most *k* hot spots for the pair *u*, *v*. Since there are at most ${|R|^2}$ pairs in *R*, the claim follows.

#### Definition 1

A hot spot ${u\in H}$ is *good* if the congestion on any edge incident on *u* is bounded by ${12 \log ({k}|R|)/\log \log ({k}|R|)}$; otherwise, *u* is *bad*.

#### Lemma 3

Let ${u\in H}$ be a hot spot. The probability that *u* is bad is bounded from above by ${1/(k^2|R|^3)}$.

#### Proof

Let ${e_1=uv_1,\dots ,e_{\ell }=uv_{\ell }}$ be the edges incident on *u* and, for each *i* with ${1\le i \le \ell }$, let ${f_i}$ be the total flow on the edge ${uv_i}$. Since any flow path visits at most two of the edges incident on *u*, the total flow ${\sum _{i=1}^{\ell }f_i}$ on the edges incident on *u* is at most 2*k*.

For any *i* with ${1 \le i \le \ell }$, we have ${f_i=\sum _{P:P\ni {e_i}}f(P)}$, where *P* runs over the set of all paths connecting some terminal pair and containing ${e_i}$. For ${1 \le j \le k}$, we define

$$\begin{aligned} {f_{ij}=\sum _{P\in \mathcal {P}(s_j,t_j):P\ni e_i}f(P)} \end{aligned}$$

as the total amount of flow sent across ${e_i}$ by the terminal pair ${(s_j,t_j)}$. Recall that ${x_j}$ is the total flow sent for the terminal pair ${(s_j,t_j)}$. The probability that the randomized rounding procedure picks a certain path ${P\in \mathcal {P}(s_j,t_j)}$ is precisely $x_j\cdot \left( f(P)/x_j\right) =f(P)$. Given the disjointness of the respective events, the probability that the pair ${(s_j,t_j)}$ routes a path across ${e_i}$ is precisely ${f_{ij}}$. Let ${X_{ij}}$ be the binary random variable indicating whether the pair ${(s_j,t_j)}$ routes a path across ${e_i}$. Then ${\mathbb {P}\left[ {X_{ij}=1}\right] =f_{ij}}$. Let ${X_i=\sum _{j=1}^{k}X_{ij}}$ be the number of paths routed across ${e_i}$ by the algorithm. By linearity of expectation,

$$\begin{aligned} \mathbb {E}\left[ {X_i}\right] =\sum _{j=1}^{k}\mathbb {E}\left[ {X_{ij}}\right] =\sum _{j=1}^{k}f_{ij}=f_i. \end{aligned}$$

In the following, we assume that *k* is sufficiently big ( ${k\ge e^{e^e}}$). Note that this assumption is feasible as MaxEDP can be efficiently solved when *k* is constant [42]. Fix any edge ${e_i}$. Set

$$\begin{aligned} {\delta =6\cdot \frac{\log ({k}|R|)}{\log {\log ({k}|R|)}}} \end{aligned}$$

and ${\delta '=2 \delta /f_i -1}$. Note that, for fixed *i*, the variables in $\{{X_{ij}}\mid 1\le j \le k\}$ are independent. Hence, by the Chernoff bound, we have

1 $$\begin{aligned} \mathbb {P}\left[ {X_i\ge {2\delta }}\right]&\le \mathbb {P}\left[ {X_i\ge (1+\delta ')f_i}\right] < \left( \frac{e^{\delta '}}{(1+\delta ')^{1+\delta '}}\right) ^{f_i}\nonumber \\&\le \left( \frac{f_i}{2}\right) ^{2\delta }\cdot ~\left( \frac{e}{\delta }\right) ^{2\delta }\nonumber \\&\le \frac{f_i}{2} \cdot e^{- 6 \cdot \frac{\log ({k}|R|)}{\log {\log ({k}|R|)}} \log \left( \frac{\log ({k}|R|)}{\log {\log ({k}|R|)}}\right) } \end{aligned}$$

2 $$\begin{aligned}&\le \frac{f_i}{2k^3|R|^3}. \end{aligned}$$

For Eq. 1, we use ${f_i\le 2}$ (see Lemma 1) and ${e/\delta \le \delta ^{-1/2}}$. For Eq. 2, we use

$$\begin{aligned} \frac{\log {\log {\log ({k}|R|)}}}{\log {\log ({k}|R|)}} \le \frac{1}{e} < \frac{1}{2}. \end{aligned}$$

Now, applying the union bound, we can infer that the probability that any of the edges incident on *u* carries more than ${{2}\delta }$ paths, that is, more than ${12 \log ({k}|R|)/\log \log ({k}|R|)}$ paths, is at most

$$\begin{aligned} \sum _{i} \frac{f_i}{2k^3|R|^3}\le \frac{2k}{2k^3|R|^3}=\frac{1}{k^2|R|^3}. \end{aligned}$$

$$\square $$

#### Lemma 4

If every hot spot is good, then the congestion on every edge is bounded from above by ${{24}\log ({k}|R|)/\log \log ({k}|R|)}$.

#### Proof

Consider an arbitrary edge ${e=uv}$ that is not incident on any hot spot. In particular, this means that *e* lies in the forest ${F=G-R}$. A hot spot *z* in *F* is called *direct* to *e* if the path in *F* from *z* to *e* excluding *e* does not contain any hot spot other than *z*.

We claim that there can be at most two distinct hot spots ${z,z'}$ direct to *e*. If there were a third hot spot ${z''}$ direct to *e*, then consider the unique node ${z_0\in V(F)}$ such that no two of the hot spots *z*, $z'$, $z''$ are connected in ${F-z_0}$. Such a node ${z_0}$ exists, since *z*, $z'$, $z''$ cannot lie on a common path in *F* as they are all direct to *e*. The node ${z_0}$, however, would be added as a hot spot at the latest when ${H}$ was built. Now this is a contradiction, because then one of the paths connecting *z*, $z'$ or ${z''}$ to *e* would contain ${z_0}$ and thus one of these hot spots would not be direct to *e*.

Now we show the lemma assuming that there are two distinct hot spots ${z,z'}$ direct to *e*. If there were only one or no hot spot direct to *e*, then we can apply a similar argument as the following one.

**Fig. 3:**
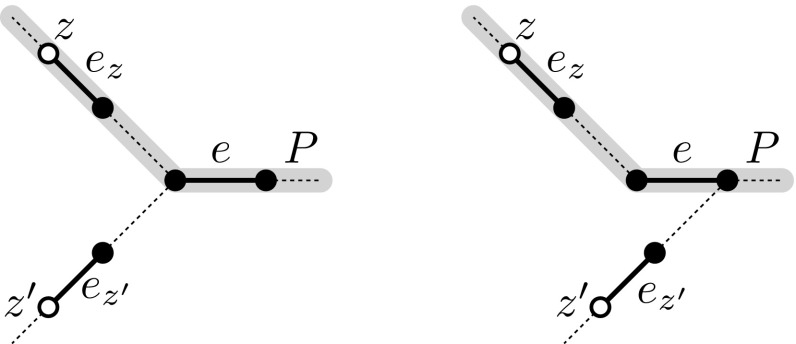
Two examples of an edge *e* with two hot spots *z* and ${z'}$ (white nodes) being direct to *e*. Note that there is no hot spot in between *z* and ${z'}$. Any path routed by our algorithm that visits *e* must visit ${e_z}$ or ${e_{z'}}$. Such a path *P* is highlighted in gray

Now, let *P* be an arbitrary path that is routed by our algorithm and that traverses *e*, and let ${P'\in \mathcal {S}}$ be the subpath of *P* visiting *e*; see Fig. 3.

Consider the two paths in *F* connecting *z* to *e* and ${z'}$ to *e*. Let ${e_z}$ and ${e_{z'}}$ be the edges on these paths incident on *z* and ${z'}$, respectively. By our construction, $P'$ must visit a hot spot in *F*. If ${P'}$ visited neither *z* nor ${z'}$, then ${P'}$ would contain a hot spot direct to *u* or to *v* that is distinct from *z* and ${z'}$—a contradiction. Hence ${P'}$ and thus also *P* visit ${e_z}$ or ${e_{z'}}$. The claim now follows from the facts that, first, this holds for any path traversing *e*, and that, secondly, *z* and ${z'}$ are good, and that, thirdly, therefore altogether at most ${2\cdot ({12}\log ({k}|R|)/\log \log ({k}|R|)}$ paths visit ${e_z}$ or ${e_z'}$.

Now we are ready to prove Theorem 1.

#### Proof of Theorem 1

We show that the algorithm presented in Sect. 3.1 produces—with constant probability—a routing with $${\varOmega ({\text {OPT}}^{*})}$$ paths with congestion $$\mathcal {O}\left( {\log ({k}r)}/{\log \log ({k}r)}\right) $$. As argued above, the probability that we route less than $${{\text {OPT}}^{*}\!\!/2}$$ paths is at most 1 / 2. By Lemma 2, the number of hot spots is at most $$2k|R|^2+|R| \le $$ $$ 3k|R|^2$$. Thus, Lemma 3 implies an upper bound of $$3k|R|^2/(k^2|R|^3)=3/(k|R|)$$ on the probability that at least one of these hot spots is bad. Hence, by Lemma 4, we route with probability $${1 - 1/2 - 3/(k|R|)}$$ at least $${{\text {OPT}}^{*}\!\!/2}$$ pairs with congestion at most $${{24}\log ({k}|R|)/\log \log ({k}|R|)}$$. Since the probability is bounded from below by a positive constant for sufficiently big *k*, the statement of the theorem follows by using $${|R|\le 2r+2k}$$ and $${|R|\ge r}$$.

## Refined approximation bound for MaxEDP

In this section, we provide an improved approximation guarantee for MaxEDP *without* congestion, thereby proving Theorem 2.

### Irreducible routings with low congestion

We first develop the concept of *irreducible routings with low congestion*, which is (besides Theorem 1) a key ingredient of our strengthened bound on the approximability of MaxEDP based on feedback vertex set number.

Consider any multigraph *G* and any set ${\mathcal {P}}$ of (not necessarily simple) paths in *G* with congestion *c*. We say that an edge *e* is *redundant in* ${\mathcal {P}}$ if there is an edge ${e'\ne e}$ such that the set of paths in ${\mathcal {P}}$ *covering* (containing) *e* is a subset of the set of paths in ${\mathcal {P}}$ covering ${e'}$. For instance, if *G* contains at least two edges, then any edge that is not covered by any path in ${\mathcal {P}}$ is redundant in ${\mathcal {P}}$.

#### Definition 2

The set ${\mathcal {P}}$ is called an *irreducible routing with congestion c* if each edge belongs to at most *c* paths of ${\mathcal {P}}$ and there is no edge redundant in ${\mathcal {P}}$.

In contrast to a feasible routing of a MaxEDP instance, we do not require an irreducible routing to connect a set of terminal pairs. If there is an edge *e* redundant in ${\mathcal {P}}$, we can apply the following *reduction rule*: we contract *e* in *G* and we contract *e* in every path of ${\mathcal {P}}$ that covers *e*. By this, we obtain a minor ${G'}$ of *G* and a set ${\mathcal {P}'}$ of paths that consists of all the contracted paths and of all paths in ${\mathcal {P}}$ that were not contracted. Thus, there is a one-to-one correspondence between the paths in ${\mathcal {P}}$ and ${\mathcal {P}'}$.

We make the following observation about ${\mathcal {P}}$ and ${\mathcal {P}'}$.

#### Observation 1

A subset of paths in ${\mathcal {P}'}$ is edge-disjoint in ${G'}$ if and only if the corresponding subset of paths in ${\mathcal {P}}$ is edge-disjoint in *G*.

As applying the reduction rule strictly decreases the number of redundant edges, an iterative application of this rule yields an irreducible routing on a minor of the original graph.

#### Theorem 6

Let ${\mathcal {G}}$ be a minor-closed class of multigraphs and let ${p_{\mathcal {G}}}$ be a positive integer. If for each graph ${G\in \mathcal {G}}$ and every non-empty irreducible routing ${\mathcal {P}}$ on *G* with congestion *c* there exists a path in ${\mathcal {P}}$ of length at most ${p_{\mathcal {G}}}$, then the average length of the paths in ${\mathcal {P}}$ is at most ${c\cdot p_{\mathcal {G}}}$.

#### Proof

Take a path ${P_0}$ of length at most ${p_{\mathcal {G}}}$. Contract all edges of ${P_0}$ in *G* and obtain a minor ${G'\in \mathcal {G}}$ of *G*. For each path in ${\mathcal {P}}$ contract all edges shared with ${P_0}$ to obtain a set ${\mathcal {P}'}$ of paths. Remove ${P_0}$ along with all degenerated paths from ${\mathcal {P}'}$, thus ${|\mathcal {P}'| < |\mathcal {P}|}$. Note that ${\mathcal {P}'}$ is an irreducible routing on ${G'}$ with congestion *c*. We repeat this reduction procedure recursively on ${G'}$ and ${\mathcal {P}'}$ until ${\mathcal {P}'}$ is empty; this happens after at most ${|\mathcal {P}|}$ steps. At each step, we decrease the total path length by at most ${c\cdot p_{\mathcal {G}}}$. Hence, the total length of paths in ${\mathcal {P}}$ is at most ${|\mathcal {P}| \cdot c \cdot p_{\mathcal {G}}}$.

As a consequence of Theorem 6, we get the following result for forests.

#### Lemma 5

Let *F* be a forest and let ${\mathcal {P}}$ be a non-empty irreducible routing on *F* with congestion *c*. The average path length in ${\mathcal {P}}$ is at most 2*c*.

#### Proof

We show that ${\mathcal {P}}$ contains a path of length at most 2. Then the lemma follows immediately by applying Theorem 6 and using the fact that (simple) forests are minor-closed.

Take any tree in *F*, root it with any node and consider a leaf *v* of maximum depth. If *v* is adjacent to the root, then the tree is a star and every path in the tree has length at most 2. Otherwise, let ${e_1}$ and ${e_2}$ be the first two edges on the path from *v* to the root. By the definition of irreducible routing, the set of all paths covering ${e_1}$ is not a subset of the paths covering ${e_2}$; hence, $e_1$ is covered by a path which does not cover ${e_2}$. Since all other edges incident to ${e_1}$ end in a leaf, this path has length at most 2.

Note that the bound provided in Lemma 5 is actually tight up to a constant. Let *c* be an arbitrary integer greater than one. Consider a graph that is a path of length ${c-1}$ with a star of ${c-1}$ leaves attached to one of its endpoints. The ${c-1}$ paths of length *c* together with the ${2c-2}$ paths of length 1 form an irreducible routing with congestion *c*. The average path length is

$$\begin{aligned} \frac{(c-1)c+(2c-2)}{3c-3}=\frac{c+2}{3}. \end{aligned}$$

### Approximation algorithm

Consider an instance ${(G,\mathcal {M})}$ of MaxEDP with *k* terminal pairs. Let *R* be a 2-approximate minimum feedback vertex set in *G*; recall that we can obtain *R* in polynomial time [6]. Furthermore, let ${c =\mathcal {O}\left( \log ({k}r)/\log \log ({k}r)\right) }$ be the bound on the congestion of our algorithm in Theorem 1.

We solve the corresponding MaxEDP LP and obtain an optimal extreme point solution ${(f,\mathbf {x})}$ of total flow ${|f|={\text {OPT}}^{*}}$. By the same argument as in Sect. 3, the number of all paths with a positive flow value is polynomially bounded in the input size. Let ${\rho =\sqrt{|R|}/c}$ and let ${\mathcal {P}}$ be the set of all paths with a positive flow value that visit at most ${\rho }$ nodes of *R*.

Below we argue how to use *R*, ${\mathcal {P}}$ and *f* to obtain a feasible routing of $\varOmega \left( |f|/(c \sqrt{|R|})\right)$ paths. This routing yields an overall approximation ratio of  ${\mathcal {O}\left( \sqrt{r}\log (kr)\right) }$ and will prove Theorem 2.

We distinguish the following two cases.

*Case 1* The total flow of ${\mathcal {P}}$ is at least |*f*| / 2. We compute a new flow ${(f',\mathbf {x'})}$, where we set ${f'(P)=f(P)}$ for every path *P* in ${\mathcal {P}}$, and ${f'(P)=0}$ for any other path *P*. Thus, we have ${|f'|\ge |f|/2}$. By applying our algorithm of Sect. 3 on ${(f',\mathbf {x'})}$, we efficiently compute with constant probability a routing ${\overline{\mathcal {P}}}$ with congestion *c* containing ${\varOmega (|f'|)=\varOmega (|f|)}$ paths. Note that all paths in ${\overline{\mathcal {P}}}$ visit at most ${\rho }$ nodes of *R*. Initialize ${\mathcal {P}'}$ with ${\overline{\mathcal {P}}}$. As long as there is an edge *e* not adjacent to *R* that is redundant in ${\mathcal {P}'}$, we iteratively apply the reduction rule (see Sect. 4.1) on *e* by contracting *e* in the graph as well as in every path that covers it. Let ${G'}$ be the obtained minor of *G* with forest ${F'=G'-R}$.

Note that $F'$ is simple (in contrast to $G'$ that might contain multiple edges) as we contracted edges only in the (simple) forest $G-R$. The obtained set ${\mathcal {P}'}$ is a set of (not necessarily simple) paths in ${G'}$ corresponding to ${\overline{ \mathcal {P}}}$. In order to obtain a feasible routing for ${(G,\mathcal {M})}$ of size ${\varOmega \left( |f|/( c \rho ) \right) }$, it suffices by iterated application of Observation 1 to ${\overline{\mathcal {P}}}$ and ${\mathcal {P}'}$ that we efficiently find a subset ${\mathcal {P}_{\text {Sol}}'\subseteq {\mathcal {P}'}}$ of pairwise edge-disjoint paths of size ${|\mathcal {P}_{\text {Sol}}'| =\varOmega \left( |\overline{\mathcal {P}}|/( c \rho )\right) }$.

To obtain ${\mathcal {P}_{\text {Sol}}'}$, we first bound the total path length in ${\mathcal {P}'}$. Removing *R* from ${G'}$ “decomposes” the set ${\mathcal {P}'}$ into a set $\mathcal {S}$ of subpaths lying in ${F'}$, that is,

$$\begin{aligned} \mathcal {S}=\{S\text { is a connected component of }P\cap F'\mid P\in \mathcal {P}'\}. \end{aligned}$$

Observe that ${\mathcal {S}}$ is an irreducible set of ${F'}$ with congestion *c*, as the reduction rule is not applicable anymore. (Note that a single path in ${\mathcal {P'}}$ may lead to many paths in the cover ${\mathcal {S}}$ which are considered distinct.) Thus, by Lemma 5, the average path length in ${\mathcal {S}}$ is at most 2*c*.

Let *P* be an arbitrary path in ${\mathcal {P}'}$. Each edge on *P* that is *not* in a subpath in ${\mathcal {S}}$ is incident on a node in *R*, and each node in *R* is incident on at most two edges in *P*. Together with the fact that *P* visits less than ${\rho }$ nodes in *R*, there are less than ${2\rho }$ edges of *P* outside ${\mathcal {S}}$. By the same fact, *P* contributes at most ${\rho }$ subpaths to ${\mathcal {S}}$. Given that the average length of the subpaths in ${\mathcal {S}}$ is at most 2*c*, we can upper bound the total path length ${\sum _{P\in \mathcal {P'}}|P|}$ by ${|\mathcal {P'}| \rho (2c+2)}$. Let ${\mathcal {P}''}$ be the set of the ${|\mathcal {P}'|/2}$ shortest paths in ${\mathcal {P}'}$. Hence, each path in ${\mathcal {P}''}$ has length at most ${4 \rho (c+1)}$.

We greedily construct a feasible solution ${\mathcal {P}_{\text {Sol}}'}$ by iteratively picking an arbitrary path *P* from ${\mathcal {P}''}$, adding it to ${\mathcal {P}_{\text {Sol}}'}$ and removing all paths from ${\mathcal {P}''}$ that share some edge with *P* (including *P* itself). We stop when ${\mathcal {P}''}$ is empty. As ${\mathcal {P}''}$ has congestion *c*, we remove at most ${4\rho c(c+1)}$ paths from ${\mathcal {P}''}$ per iteration. Thus,

$$\begin{aligned} |\mathcal {P}_{\text {Sol}}'|\ge |\mathcal {P''}|/(4\rho c(c+1))=\varOmega \left( |\overline{\mathcal {P}}|/( c \sqrt{|R|})\right) . \end{aligned}$$

*Case 2* The flow of ${\mathcal {P}}$ is less than |*f*| / 2. Then, the flow of all paths visiting at least ${\rho }$ nodes of *R* is at least |*f*| / 2. Let ${\mathcal {P}'}$ be the subset of these paths and let ${f'}$ be the sum of all these flows. Note that ${f'}$ provides a feasible solution to relaxation MaxEDP LP for (*G*, *M*) of value at least |*f*| / 2. Since every flow path in ${f'}$ has length at least ${\rho }$, the total inflow of the nodes in *R* is at least ${|f'|\rho }$. By averaging, there must be a node ${v\in {R}}$ of inflow at least ${\rho |f'|/{|R|} = |f'|/(c\sqrt{|R|})}$. Let ${f''}$ be the subflow of ${f'}$ consisting of all flow paths visiting *v*. This subflow corresponds to a feasible solution ${(f'',\mathbf {x''})}$ of the LP relaxation of value at least ${ |f'|/(c\sqrt{|R|}) \ge |f|/(2c\sqrt{|R|})}$. Using Proposition 1, we can recover an integral feasible routing of size at least

$$\begin{aligned} \sum _{i}x_i''/12 \ge |f|/(24c\sqrt{|R|}) =\varOmega \left( |f|/( c \sqrt{|R|}\right) . \end{aligned}$$

This completes the proof of Theorem 2. $\square$

## Fixed-parameter algorithm for MaxNDP

We give a fixed-parameter algorithm for MaxNDP that solves anyinstance ${(G,\mathcal {M})}$ in time ${(k+r)^{\mathcal {O}(r)}\cdot ~n}$, where *r* denotes the feedback vertex set number of *G*, $k=|\mathcal {M}|$ and ${n = |V(G)|}$. A feedback vertex set *R* of size *r* can be computed in time ${r^{\mathcal {O}(r)}\cdot n}$ [38].

By the matching assumption (see Sect. 2), each terminal in ${\mathcal {M}}$ is a leaf. We can thus assume that none of the terminals is contained in *R*.

Consider an optimal routing ${\mathcal {P}}$ of the given MaxNDP instance and the set ${\mathcal {M}_R\subseteq \mathcal {M}}$ of terminal pairs that are connected via ${\mathcal {P}}$ by a path that visits at least one node in *R*. Let ${P\in \mathcal {P}}$ be a path connecting a terminal pair ${(s_i,t_i)\in \mathcal {M}_R}$. This path has the form ${(s_i,\dots ,r_1,\dots ,r_2,\dots ,r_\ell ,\dots ,t_i)}$, where ${r_1,\dots ,r_\ell }$ are the nodes in *R* that are traversed by *P* in this order. The pairs ${(s_i,r_1)}$ and ${(r_\ell ,t_i)}$ as well as ${(r_j,r_{j+1})}$ for ${j=1,\dots ,\ell -1}$ are called *essential* pairs *for* *P*. A node pair is called *essential* if it is essential for some path in ${\mathcal {P}}$. Let ${\mathcal {M}_e}$ be the set of essential pairs.

Let *F* be the forest that arises when deleting *R* from the input graph *G*. Let (*u*, *v*) be any pair of nodes in *G*. A path *P* in *G* with endpoints *u* and *v* is said to *realize* (*u*, *v*) if all internal nodes of *P* lie in *F*. A set ${\mathcal {P}'}$ of paths is said to *realize* a set of node pairs if every pair in this set is realized by some path in ${\mathcal {P}'}$ and if two paths in ${\mathcal {P}'}$ can only intersect at their endpoints. Note that the optimal routing ${\mathcal {P}}$ induces a realization of ${\mathcal {M}_e}$ in a natural way: The realization consists of all maximal subpaths of paths in ${\mathcal {P}}$ whose internal nodes all lie in *F*. Conversely, for any realization ${\mathcal {P}'}$ of ${\mathcal {M}_e}$, we can concatenate paths in ${\mathcal {P}'}$ to obtain a feasible routing that connects all terminal pairs in ${\mathcal {M}_R}$. Therefore, we consider ${\mathcal {P}'}$ (slightly abusing notation) also as a feasible routing for ${\mathcal {M}_R}$.

In our algorithm, we first guess the set ${\mathcal {M}_e}$ of essential pairs, which implies the set ${\mathcal {M}_R}$ as well as the set ${\overline{\mathcal {M}}_R}$ that we define as ${\overline{\mathcal {M}}_R=\mathcal {M}{\setminus }\mathcal {M}_R}$. Then, by dynamic programming, we construct two sets of paths, ${\mathcal {P}_e}$ and ${\mathcal {P}_F}$, where ${\mathcal {P}_e}$ realizes ${\mathcal {M}_e}$ and ${\mathcal {P}_F}$ routes in *F* a subset of ${\overline{\mathcal {M}}_R}$. In our algorithm, the set ${\mathcal {P}_e \cup \mathcal {P}_F}$ forms a feasible routing that maximizes ${|\mathcal {P}_F|}$ and routes all pairs in ${\mathcal {M}_R}$. Recall that we consider the realization ${\mathcal {P}_e}$ of ${\mathcal {M}_e}$ as a feasible routing for ${\mathcal {M}_R}$.

Now assume that we correctly guessed ${\mathcal {M}_e}$. Below, we will describe an algorithm that uses a dynamic programming table to compute an optimum routing in time ${2^{\mathcal {O}(r)}(k+r)^{\mathcal {O}(1)}\cdot n}$. For the sake of easier presentation, first we describe how to compute the cardinality of such a routing. Then we argue how to find such a routing without a significant increase in the run time.

### Dynamic programming table

Before we describe the dynamic programming table, we make several technical assumptions that help to simplify the presentation. First, we modify the input instance as follows. We subdivide every edge incident on a node in *R* by introducing a single new node on this edge. Note that this yields an instance equivalent to the input instance. As a result, every neighbor of a node in *R* that lies in *F*, that is, every node in ${N_G(R)}$, is a leaf in *F*. Moreover, the set *R* is an independent set in *G*. Also recall that we assumed that every terminal is a leaf and that therefore *R* does not contain any terminal. We also assume that the forest *F* is a rooted tree by introducing a dummy node (which plays the role of the root) and arbitrarily connecting this node to every connected component of *F* by an edge. In our dynamic programming table, we will take care that no path visits this root node. We also assume that *F* is an ordered tree by introducing an arbitrary order among the children of every node.

**Fig. 4:**
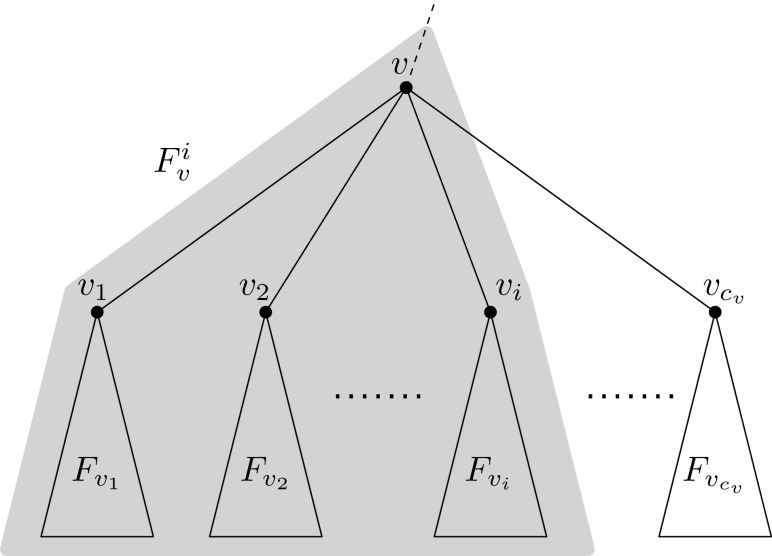
Subtree ${F_v^i}$ consists of *v* and subtrees ${F_{v_1},\dots ,F_{v_i}}$. Recall that only leaf nodes can be terminals or neighbors of *R*

For any node *v*, let ${F_v}$ be the subtree of *F* rooted at *v*. Let ${c_{v}}$ be the number ${\deg _F(v)-1}$ of children of *v* and let ${v_1,\dots v_{c_{v}}}$ be the (ordered) children of *v*. Then, for ${i=1,\dots ,c_{v}}$, let ${F_v^i}$ denote the subtree of ${F_v}$ induced by the union of *v* with the subtrees ${F_{v_1},\dots ,F_{v_i}}$; see Fig. 4. If *v* is a leaf, we have ${F_v=v}$ and we define ${F_v^0}$ as ${F_v}$.

We introduce a dynamic programming table *T*. It contains an entry for every ${F_v^i}$ and every subset ${\mathcal {M}_e'}$ of ${\mathcal {M}_e}$. Roughly speaking, the value of such an entry is the solution to the subproblem, where we restrict the forest to ${F_v^i}$, and the set of essential pairs to ${\mathcal {M}_e'}$. More precisely, table *T* contains five parameters: Parameters *v* and *i* describing ${F_v^i}$, a parameter ${\mathcal {M}_e'}$ describing the set of essential pairs, and two more parameters *u* and *b*. The parameter *u* is either a terminal or a node in *R*, and *b* is in one of the three states: ${\textit{free}}$, ${\textit{to-be-used}}$, or ${{\textit{blocked}}}$. The value ${T[v,i,\mathcal {M}_e',u,b]}$ is the maximum cardinality of a set ${\mathcal {P}_F}$ of paths with the following properties:

1. The set ${\mathcal {P}_F}$ is a feasible routing of some subset of ${\overline{\mathcal {M}}_R}$.
2. The set ${\mathcal {P}_F}$ is completely contained in ${F_v^i}$.
3. There is an additional set ${\mathcal {P}_e}$ of paths with the following properties:
  - The set ${\mathcal {P}_e}$ is a realization of ${\mathcal {M}_e'\cup \{(u,v)\}}$ if ${b={\textit{to-be-used}}}$. Else, it is a realization of ${\mathcal {M}_e'}$.
  - The set ${\mathcal {P}_e}$ is completely contained in ${F_v^i\cup R}$ and node-disjoint from the paths in ${\mathcal {P}_F}$.
4. If ${b={\textit{free}}}$, there is no path in ${\mathcal {P}_e\cup \mathcal {P}_F}$ visiting *v*.

If no such set ${\mathcal {P}_F}$ exists, then ${T[v,i,\mathcal {M}_e',u,b]}$ is ${-\infty }$.

Note that the parameter *u* is only relevant when ${b={\textit{to-be-used}}}$ (otherwise, it can just be ignored). One can think of the three states of *b* as follows: If ${b={\textit{free}}}$, then there is no path in ${\mathcal {P}_e\cup \mathcal {P}_F}$ visiting *v*, hence, in the future we might consider to add a path through *v*. If ${b={\textit{to-be-used}}}$, then *v* is visited by some path in ${\mathcal {P}_e}$ (connecting *u* to *v*) and we cannot add a new path through *v*. Eventually, if ${b={\textit{blocked}}}$, we may add a path to ${\mathcal {P}_e\cup \mathcal {P}_F}$ that goes through *v*. Hence, *v* is “blocked” for the future because of the possibility of having been already visited. Thus, we have

$$\begin{aligned} T[v,i,\mathcal {M}_e',u,{\textit{blocked}}]\ge T[v,i,\mathcal {M}_e',u,{\textit{free}}]\ge T[v,i,\mathcal {M}_e',u,{\textit{to-be-used}}]. \end{aligned}$$

Below, we describe how to compute the entries of *T* in a bottom-up manner. Having computed *T*, we obtain the cardinality of the optimum routing ${\mathcal {P}}$ by ${|\mathcal {M}_R| + T[v,c_{v},\mathcal {M}_e,u,{\textit{free}}]}$, where *v* is the dummy root node and *u* is an arbitrary terminal.

*Base case* In the base case, the node *v* is a leaf and we have ${\mathcal {P}_F=\emptyset }$. Thus, every entry for *v* has value either 0 or ${-\infty }$, depending on whether ${\mathcal {M}_e}$ can be routed. When ${b={\textit{free}}}$, no path can visit *v* and, hence, also ${\mathcal {P}_e=\emptyset }$. Thus we set

$$\begin{aligned} T[v,0,\emptyset ,u,{\textit{free}}]=0. \end{aligned}$$

Then we set

$$\begin{aligned} {T[v,0,\mathcal {M}_e',u,{\textit{blocked}}]=0} \end{aligned}$$

if ${\mathcal {M}_e'}$ is either empty, or consists of a single pair of nodes in ${R\cap N_G(v)}$, or consists of a single pair where one node is *v* and the other one is in ${R\cap N_G(v)}$. Finally, we set

$$\begin{aligned} T[v,0,\emptyset ,u,{\textit{to-be-used}}]=0 \end{aligned}$$

if ${u=v}$ or *u* is in ${R\cap N_G(v)}$. For all the other cases where *v* is a leaf, we set

$$\begin{aligned} T[v,i,\mathcal {M}_e',u,b]=-\infty . \end{aligned}$$

*Induction step* For the inductive step, we first consider ${i=1}$. We have

$$\begin{aligned}T[v,1,\mathcal {M}_e',u,{\textit{to-be-used}}]=T[v_1,c_{v},\mathcal {M}_e',u,{\textit{to-be-used}}],\end{aligned}$$

since the path in ${\mathcal {P}_e}$ realizing (*u*, *v*) has to start at a leaf node of ${F_{v_1}}$. For the other states of *b*, recall that every path in ${\mathcal {P}_e\cup \mathcal {P}_F}$ connects two leaves in ${F_v^1}$. Since *v* has degree 1 in ${F_v^1}$, there is no path in ${\mathcal {P}_e\cup \mathcal {P}_F}$ visiting *v*, and we have

$$\begin{aligned} T[v,1,\mathcal {M}_e',u,{\textit{blocked}}] = T[v,1,\mathcal {M}_e',u,{\textit{free}}] = T[v_1,c_{v},\mathcal {M}_e',u,{\textit{blocked}}]. \end{aligned}$$

Now, let *i* be greater than 1. In a high level view, we guess which part of ${\mathcal {M}_e'}$ is realized in ${F_{v}^{i-1}\cup R}$ and which part is realized in ${F_{v_i}\cup R}$. For this, we consider every partition ${\mathcal {M}_{e1}' \uplus \mathcal {M}_{e2}'}$ of ${\mathcal {M}_e'}$. By our dynamic programming table, we find a partition that maximizes our objective. In the following, we assume that we guessed ${\mathcal {M}_{e1}' \uplus \mathcal {M}_{e2}'}$ correctly. Let us consider the different states of *b* in more detail.

1. When ${b={\textit{free}}}$, node *v* is not allowed to be visited by any path, especially by any path in ${F_{v}^{i-1}\cup R}$. Hence, ${T[v,i,\mathcal {M}_e',u,{\textit{free}}]}$ is equal to
  $$\begin{aligned} T[v,i-1,\mathcal {M}_{e1}',u,{\textit{free}}] + T[v_i,c_{v_i},\mathcal {M}_{e2}',u,{\textit{blocked}}]. \end{aligned}$$
2. When ${b={\textit{to-be-used}}}$, we have to realize (*u*, *v*) in ${F_{v}^{i}\cup R}$. For this, there are two possibilities: Either (*u*, *v*) is realized by a path in ${F_{v}^{i-1}\cup R}$, or there is a realizing path that first goes through ${F_{v_i}\cup R}$ and then reaches *v* via the edge ${(v_i, v)}$. Hence, for the first possibility, we consider
  $$\begin{aligned} T[v,i-1,\mathcal {M}_{e1}',u,{\textit{to-be-used}}] + T[v_i,c_{v_i},\mathcal {M}_{e2}',u,{\textit{blocked}}], \end{aligned}$$
  for the second possibility, we consider
  $$\begin{aligned} T[v,i-1,\mathcal {M}_{e1}',u,{\textit{free}}] + T[v_i,c_{v_i},\mathcal {M}_{e2}',u,{\textit{to-be-used}}]. \end{aligned}$$
  Maximizing over both, we obtain ${T[v,i,\mathcal {M}_e',u,{\textit{to-be-used}}]}$.
3. When ${b={\textit{blocked}}}$, we will also consider two cases. In the first one, there is no path in ${\mathcal {P}_e\cup \mathcal {P}_F}$ going through edge ${(v_i,v)}$, hence, we get the term
  $$\begin{aligned} T[v,i-1,\mathcal {M}_{e1}',u,{\textit{blocked}}] + T[v_i,c_{v_i},\mathcal {M}_{e2}',u,{\textit{blocked}}]. \end{aligned}$$
  In the second case, there is a path *P* in ${\mathcal {P}_e\cup \mathcal {P}_F}$ going through edge ${(v_i,v)}$. Since *P* is connecting two leaves in ${F_{v}^{i}}$, a part of *P* is in ${F_{v}^{i-1}\cup R}$ and the other part is in ${F_{v_i}\cup R}$. If ${P\in \mathcal {P}_e}$, then it is realizing a pair of ${\mathcal {M}_{e}'}$. Hence, for every pair ${(u_1,u_2)\in \mathcal {M}_{e}'}$, we have to consider the term
  $$\begin{aligned}&T[v,i-1,\mathcal {M}_{e1}'-(u_1,u_2),u_1,{\textit{to-be-used}}]\\&\quad +~T[v_i,c_{v_i},\mathcal {M}_{e2}'-(u_1,u_2),u_2,{\textit{to-be-used}}] \end{aligned}$$
  and the symmetric term where we swap ${u_1}$ and ${u_2}$. If ${P\in \mathcal {P}_F}$, then it is realizing a terminal pair of ${\overline{\mathcal {M}}_R}$. Hence, for every pair ${(u_1,u_2)\in \overline{\mathcal {M}}_R}$ we get the term
  $$\begin{aligned} 1+ T[v,i-1,\mathcal {M}_{e1}',u_1,{\textit{to-be-used}}] + T[v_i,c_{v_i},\mathcal {M}_{e2}',u_2,{\textit{to-be-used}}] \end{aligned}$$
  and the symmetric term where we swap ${u_1}$ and ${u_2}$. Note that we count the path realizing ${(u_1,u_2)}$ in our objective. Maximizing over all the terms of the two cases, we obtain ${T[v,i,\mathcal {M}_e',u,{\textit{to-be-used}}]}$.

### Analysis

Let us analyze the run time of the algorithm described above. Given *R*, the forest *F* can be computed in time ${\mathcal {O}(r \cdot n)}$. In order to guess ${\mathcal {M}_e}$, we enumerate all potential sets of essential pairs. To bound the number of potential sets of essential pairs, first recall that each pair contains at least one node in *R*. On the other hand, each node in *R* appears in at most two pairs and, consequently, ${|\mathcal {M}_e|\le 2r}$. Thus, an upper bound on the number of potential sets for ${\mathcal {M}_e}$ is the number of ways to choose up to two pairs for each node in *R*. As each node in *R* is paired with a terminal node or another node in *R*, there are at most ${(2k+r-1)}$ candidate pairs for it. Hence, there are at most ${(2k+r)^{2r}}$ candidate sets to consider. For each particular guess for ${\mathcal {M}_e}$, we run the dynamic program above. The number of entries in *T*—as specified by the five parameters *v*, *i*, $\mathcal {M}_e', u$ and *b*—for each fixed guess for ${\mathcal {M}_e}$ is at most

$$\begin{aligned} \left( \sum _{v\in V(F)}\deg _F(v)\right) \cdot 2^{2r}\cdot (2k+r)\cdot 3 =2^{2r} \cdot (2k+r) \cdot \mathcal {O}( n). \end{aligned}$$

Among the different entries, those with ${b={\textit{blocked}}}$ and ${i>1}$ have the highest run time in the worst case. There, we do not only consider all partitions of ${\mathcal {M}_e'}$, but for every partition we also consider every possible node pair that is either an essential pair in ${\mathcal {M}_e'}$ or a terminal pair in ${\overline{\mathcal {M}}_R}$. As there are at most ${2^{2r}}$ partitions of ${\mathcal {M}_e'}$, at most 2*r* essential pairs in ${\mathcal {M}_e'}$ and at most *k* terminal pairs in ${\overline{\mathcal {M}}_R}$, we consider at most ${2^{2r} + 2\cdot 2^{2r} \cdot (k+2r)\le 2^{2r+1} \cdot (2k+2r)}$ different terms, including the symmetric terms, for computing an entry. For each term, we need constant time for look-up. Hence, altogether, this gives a run time of

$$\begin{aligned} r \cdot (2k+r)^{2r} \cdot 2^{2r} \cdot (2k+r) \cdot 2^{2r+1} \cdot (2k+2r) \cdot \mathcal {O}(n) =(8k+8r)^{2r+3}\cdot \mathcal {O}(n) \end{aligned}$$

assuming that *R* is given. By computing *R* in time ${r^{\mathcal {O}(r)}\cdot n}$, we can bound the total run time by ${(k+r)^{\mathcal {O}(r)}\cdot n}$.

### Reconstruction of an optimal routing

Above, we computed only the cardinality of the routing ${\mathcal {P}}$. Now we discuss how to compute an optimal routing of size ${|\mathcal {P}|}$ without asymptotically increasing the total run time. For every non-leaf entry of *T*, we take a term that maximized its value and define the (at most two) entries appearing in the term as its children. We can do this while computing *T* without increasing the asymptotic run time. By considering all the children that (recursively) contributed to the entry with the optimum value of the root node, we obtain a *computation tree*. Going over the computation tree from bottom to top, we compute for each entry of the tree its set of paths ${\mathcal {P}_e\cup \mathcal {P}_F}$. We store the set as a linked list with pointers to the paths which themselves are stored as linked lists of their nodes. Whenever we concatenate two lists, we will not create a new copy but reuse one of them. This will give us constant time for concatenation. Note that for almost all entries we obtain ${\mathcal {P}_e\cup \mathcal {P}_F}$ by just taking the union of the paths of its children. Hence, we just concatenate the lists of its children (at most two) in constant time. The only exception are entries where ${b={\textit{blocked}}}$ and a path *P* is going through the node given by the first parameter *v* of the entry. Here, we obtain *P* by concatenating two paths, where each one belongs to a different child of the entry. Then we add the concatenated path to the union of the remaining paths of the children. The operation to find the two paths that we want to concatenate takes ${\mathcal {O}(|\mathcal {P}_e\cup \mathcal {P}_F|)=\mathcal {O}(k + r)}$ time. The remaining steps to compute ${\mathcal {P}_e\cup \mathcal {P}_F}$ also take constant time. Thus, for each entry of the tree, we can bound the run time by ${\mathcal {O}(k + r)}$. Note that in the computation tree there is exactly one entry for each subtree ${F_v^i}$, hence, in total there are ${\mathcal {O}(n)}$ entries. Thus, our approach takes additional time of ${(k + r) \cdot \mathcal {O}(n)}$ to compute the paths ${\mathcal {P}_e\cup \mathcal {P}_F}$. Finally, the time needed to accordingly concatenate the paths in ${\mathcal {P}_e}$ to get a routing for ${\mathcal {M}_R}$ takes at most ${\mathcal {O}(|\mathcal {P}_e|^2) =\mathcal {O}(r^2)}$ time. Hence, in time ${(8k+8r)^{2r+3}\cdot \mathcal {O}(n)}$ we can compute an optimal routing, asymptotically matching the time needed to compute its cardinality.

This finishes the proof of Theorem 3. $\square$

## Parameterized intractability of MaxNDP for the Parameter *r*

In this section, we prove Theorem 4, that is, we show that MaxNDP is ${\mathsf {W}[1]}$-hard parameterized by feedback vertex set number. This reduction was originally devised for the parameter tree-depth by Ene et al. [21]; we notice that the same reduction also works for the parameter *r*. (Both tree-depth and feedback vertex set number are restrictions of treewidth, but they are incomparable to each other.)

For sake of completeness, we include the reduction here, and argue about the feedback vertex set number of the reduced graph. The reduction is from the *W*[1]-hard Multicolored Clique problem [23], where given a graph *G* and a partition of *V*(*G*) into *q* independent sets ${V^1,\ldots ,V^q}$, we are to check if there exists a *q*-clique in *G* with exactly one vertex in every set ${V^i}$. By adding dummy vertices, we can assume ${q\ge 2}$ and ${|V^i| = n}$ for some *n* with ${n \ge 2}$ and every *i* with ${1\le i \le q}$.

*Construction* Given an instance ${(G,(V^i)_{i=1}^q)}$ of Multicolored Clique, we aim at constructing an equivalent instance ${(H,\mathcal {M},\ell )}$ of MaxNDP consisting of a graph *H* with feedback vertex set number bounded by a function of *q*, a set *M* of terminal pairs, and an integer ${\ell }$. The graph *H* will contain ${\ell }$ node-disjoint paths, each one routing a distinct terminal pair in *M*, if and only if ${(G,(V^i)_{i=1}^q)}$ is a “yes”-instance.

**Fig. 5:**
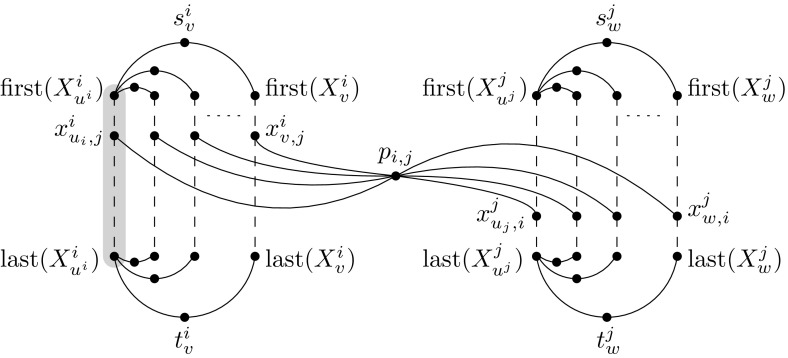
Part of the construction of the graph *H*: The gadgets ${W^i}$ and ${W^j}$ connected via ${p_{i,j}}$. On the left side, the path ${X_{u^i}^i}$ is highlighted in gray

We start by constructing for every set ${V^i}$ a gadget ${W^i}$ as follows. First, for every ${v \in V^i}$, we construct a path ${X_v^i}$ of length ${q-2}$ on the vertex set

$$\begin{aligned} {\{x_{v,j}^i \mid j \in \{1,\ldots ,q\}{\setminus }\{i\}\}}, \end{aligned}$$

where the vertices are connected in any order. Let ${{\text {first}}(X_v^i)}$ denote any one of the two endpoints of ${X_v^i}$, and let ${{\text {last}}(X_v^i)}$ denote the other endpoint of ${X_v^i}$. Secondly, we select an arbitrary vertex ${u^i \in V^i}$. Thirdly, for every ${v \in V^i {\setminus } \{u^i\}}$, we add a vertex ${s^i_v}$ and a vertex ${t^i_v}$. We make ${s^i_v}$ adjacent to ${{\text {first}}(X_v^i)}$ and to ${{\text {first}}(X_{u^i}^i)}$. Similarly, we make ${t^i_v}$ adjacent to ${{\text {last}}(X_v^i)}$ and to ${{\text {last}}(X_{u^i}^i)}$; see Fig. 5. We set ${(s^i_v,t^i_v)}$ as a terminal pair. This concludes the description of the gadget ${W^i}$. Let ${\mathcal {M}_{st}}$ denote the set of terminal pairs constructed in this step.

To encode adjacencies in *G*, we proceed as follows. For every *i* and *j* with ${1 \le i < j \le q}$, we add a vertex ${p_{i,j}}$ adjacent to all vertices in ${\{x_{v,j}^i \mid v \in V^i\}}$ and in ${\{x_{w,i}^j \mid w \in V^j\}}$; see Fig. 5. For every edge ${vw \in E(G)}$ with ${v \in V^i}$ and ${w \in V^j}$, we add a terminal pair ${(x_{v,j}^i, x_{w,i}^j)}$. Let ${\mathcal {M}_x}$ be the set of terminal pairs constructed in this step; we have ${\mathcal {M} = \mathcal {M}_{st} \cup \mathcal {M}_x}$.

Finally, we set the required number ${\ell }$ of paths to $${q(n-1) + \left( {\begin{array}{c}q\\ 2\end{array}}\right) }$$. This concludes the description of the instance ${(H,\mathcal {M},\ell )}$.

*From a clique to disjoint paths* Assume that the given instance of Multicolored Clique is a “yes”-instance, and let ${\{v^i\mid i\in \{1,\ldots ,q\} \}}$ be a clique in *G* with ${v^i \in V^i}$ for each ${i \in \{1,\ldots ,q\}}$. We construct a family of ${\ell }$ node-disjoint paths as follows. First, for every ${i \in \{1,\ldots ,q\}}$ and every ${v \in V^i {\setminus } \{u^i\}}$, we route a path from ${s^i_v}$ to ${t^i_v}$ through the path ${X_v^i}$ if ${v \ne v^i}$, and through the path ${X_{u^i}^i}$ if ${v = v^i}$. Note that in this step we have created ${q(n-1)}$ node-disjoint paths connecting terminal pairs, and in every gadget ${W^i}$ the only unused vertices are vertices on the path ${X_{v^i}^i}$. To construct the remaining $${\left( {\begin{array}{c}q\\ 2\end{array}}\right) }$$ paths, for every *i* and *j* with ${1 \le i < j \le q}$, we take the 3-vertex path from ${x_{v^i,j}^i}$ to ${x_{v^j,i}^j}$ through ${p_{i,j}}$; note that the assumption ${v^iv^j \in E(G)}$ ensures that ${(x_{v^i,j}^i, x_{v^j,i}^j)}$ is indeed a terminal pair in ${\mathcal {M}}$.

*From disjoint paths to a clique.* In the other direction, let ${\mathcal {P}}$ be a family of ${\ell }$ node-disjoint paths connecting terminal pairs in *H*. Let ${\mathcal {P}_{st} \subseteq \mathcal {P}}$ be the set of paths connecting terminal pairs from ${\mathcal {M}_{st}}$, and, in an analogous way, let ${\mathcal {P}_x\subseteq \mathcal {P}}$ be the set of paths connecting terminal pairs from ${\mathcal {M}_x}$. Eventually, let ${P=\{p_{i,j}\mid 1 \le i < j \le q\}}$. First, observe that *P* separates every terminal pair from ${\mathcal {M}_x}$. Hence, every path from ${\mathcal {P}_x}$ contains at least one vertex from *P*. Since $${|P| = \left( {\begin{array}{c}q\\ 2\end{array}}\right) }$$, we have $${|\mathcal {P}_x| \le \left( {\begin{array}{c}q\\ 2\end{array}}\right) }$$, and, consequently,

$$\begin{aligned} {|\mathcal {P}_{st}| \ge \ell - \left( {\begin{array}{c}q\\ 2\end{array}}\right) = q(n-1) = |\mathcal {M}_{st}|}. \end{aligned}$$

Thus, $\mathcal {P}_{st}$ routes all terminal pairs in ${\mathcal {M}_{st}}$ and ${\mathcal {P}_x}$ routes $${\left( {\begin{array}{c}q\\ 2\end{array}}\right) }$$ pairs from ${\mathcal {M}_x}$. Since ${|\mathcal {P}_x|=|P|}$, every vertex in *P* is contained in a path from ${\mathcal {P}_x}$. Consequently, the paths in ${\mathcal {P}_{st}}$ cannot use any vertex in *P*. Therefore, every path in ${\mathcal {P}_{st}}$ lies inside one gadget ${W^i}$.

Observe that a shortest path between terminals ${s_{v}^i}$ and ${t_{v}^i}$ inside ${W^i}$ is either ${X_{u^i}^i}$ or ${X_v^i}$ prolonged with the terminals at endpoints, and thus contains ${q+1}$ vertices. Furthermore, a shortest path between two terminals in ${\mathcal {M}_x}$ contains three vertices. We infer that the total number of vertices on paths in ${\mathcal {P}}$ is at least

$$\begin{aligned} |\mathcal {P}_{st}| \cdot (q+1) + |\mathcal {P}_x| \cdot 3&= q(n-1)(q+1) + 3\left( {\begin{array}{c}q\\ 2\end{array}}\right) \\&= q \left( (n-1)(q+1) + (q-1)\right) + \left( {\begin{array}{c}q\\ 2\end{array}}\right) = |V(H)|. \end{aligned}$$

We infer that every path in ${\mathcal {P}_{st}}$ consists of ${q+1}$ vertices, and every path in ${\mathcal {P}_x}$ consists of three vertices. In particular, for every ${i \in \{1,\ldots ,q\}}$ and every ${v \in V^i {\setminus } \{u^i\}}$, the path in ${\mathcal {P}_{st}}$ that connects ${s_v^i}$ and ${t_v^i}$ goes either through ${X_v^i}$ or ${X_{u^i}^i}$. Consequently, for each ${i \in \{1,\ldots , q\}}$ there exists a vertex ${v^i \in V^i}$ such that the path ${X_{v^i}^i}$ is not contained in any path from ${\mathcal {P}_{st}}$. Even more, ${X_{v^i}^i}$ contains all the vertices of ${W^i}$ that do not lie on any path from ${\mathcal {P}_{st}}$.

We claim that ${\{v^i\mid i=1,\ldots ,q\}}$ is a clique in *G*. To this end, consider any ${p_{i,j}\in P}$. Since ${|\mathcal {P}_x| = |P|}$, there exists a path in ${\mathcal {P}_x}$ that goes through ${p_{i,j}}$. Moreover, this path has exactly three vertices. Since the only neighbors of ${p_{i,j}}$ that are not used by paths from ${\mathcal {P}_{st}}$ are ${x_{v^i,j}^i}$ and ${x_{v^j,i}^j}$, we infer that ${(x_{v^i,j}^i, x_{v^j,i}^j)}$ is a terminal pair in ${\mathcal {M}}$ and, consequently, $v^iv^j \in E(G)$. This concludes the proof of the correctness of the construction.

*Bounding the feedback vertex set number* We are left with a proof that *H* has bounded feedback vertex set number in *q*.

First, observe that ${H-P}$ consists of *q* components, where each component is a gadget ${W^i}$, for some ${i\in \{1,\ldots , q\}}$. Secondly, consider the endpoints of the path ${X_{u^i}^i}$ from the gadget ${W^i}$. Observe that the deletion of both vertices breaks ${W^i}$ into *n* componentswhere each component is a path. Consequently, *H* has the feedback vertex set

$$\begin{aligned} P \cup \{{\text {first}}(X_{u^i}^i), {\text {last}}(X_{u^i}^i)\mid i=1,\ldots ,q\} \end{aligned}$$

of size $${\mathcal {O}(q^2)}$$.

This finishes the proof of Theorem 4. $\square$

## Hardness of edge-disjoint paths in almost-forests

In this section, we show that EDP is ${\mathsf {NP}}$-hard already in graphs that become forests after deleting two nodes. Though this immediately implies ${\mathsf {NP}}$-hardness for MaxEDP in such graphs, we show that MaxEDP is ${\mathsf {NP}}$-hard even in graphs that become forests after deleting just one node. Thus, we prove Theorem 5.

### Proof of Theorem 5

**Fig. 6:**
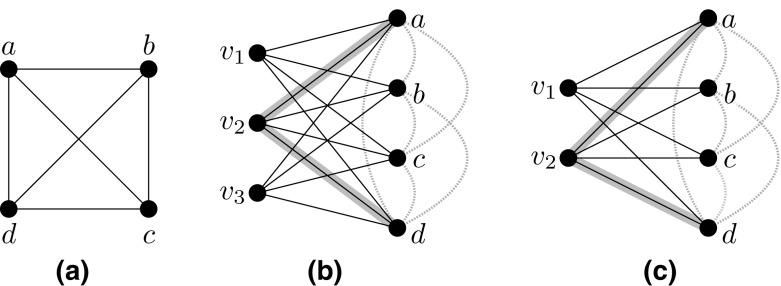
**a** Graph *H*; it is 3-edge-colorable. **b** Graph *G* obtained from *H* with ${r=2}$. **c** Graph *G* obtained from *H* with ${r=1}$. The reduction from an Edge 3-Coloring-instance *H* to an EDP/MaxEDP-instance ${(G,\mathcal {M})}$. Dotted curves depict which terminals form a pair in ${\mathcal {M}}$. The path highlighted in gray connects the terminal pair ${\{a,d\}}$

We first show ${\mathsf {NP}}$-hardness of EDP for ${r=2}$. We reduce from the problem Edge 3-Coloring in cubic graphs, which is ${\mathsf {NP}}$-hard [28]. Given a cubic graph *H*, we construct a complete bipartite graph *G*, where one of the two partite sets of *V*(*G*) consists of three nodes ${\{v_1,v_2,v_3\}}$, and the other partite set consists of *V*(*H*); see Fig. 6a, b. As for the set ${\mathcal {M}}$ of terminal pairs, let ${\mathcal {M} = \{(s,t)\mid \{s,t\}\in E(H)\}}$; in words, we want to connect a pair of nodes by a path in *G* if and only if they are connected by an edge in *H*. This completes the construction of the instance ${(G,\mathcal {M})}$ of MaxEDP. Note that *G* has feedback vertex set number ${r=2}$; removing from *G* any two vertices of ${\{v_1,v_2,v_3\}}$ yields a forest.

Regarding correctness of the reduction, we show that *H* is 3-edge-colorable if and only if *all* pairs in ${\mathcal {M}}$ can be routed in *G*.

In the forward direction, suppose that *H* is 3-edge-colorable by a proper coloring ${\varphi :E(H)\rightarrow \{1,2,3\}}$. For ${c \in \{1,2,3\}}$, let ${E_c\subseteq E(H)}$ be the set of edges that receive color *c* under ${\varphi }$. Then there is a routing in *G* that, for every ${c \in \{1,2,3\}}$, routes all terminal pairs ${\{(s,t)\in \mathcal {M}\mid \{s,t\}\in E_c\}}$ exclusively via the node ${v_c}$ (and thus via paths of length 2). Note that this routing indeed yields edge-disjoint paths. Otherwise there were an edge ${\{s,v_c\}}$ in *E*(*H*) contained in at least two paths that route two terminal pairs ${\{s,t_1\}}$ and ${\{s,t_2\}}$. Hence, the two edges in *E*(*H*) corresponding to ${\{s,t_1\}}$ and ${\{s,t_2\}}$ would receive the same color *c* in ${\varphi }$; a contradiction to the proper edge-coloring ${\varphi }$ as both edges are incident on *s*.

In the backward direction, suppose that all terminal pairs in ${\mathcal {M}}$ can be routed in *G*. Since *H* is cubic, any node ${s\in V(H)}$ is contained in three terminal pairs. Therefore, no path of the routing can have a node in *V*(*H*) as an internal node and thus all paths in the routing have length 2. Then this routing naturally corresponds to a proper 3-edge-coloring ${\varphi }$ of *H*, where any terminal pair ${\{s,t\}}$ routed via ${v_c\in \{v_1,v_2,v_3\}}$ means that we color the edge ${\{s,t\}\in E(H)}$ with color *c* under ${\varphi }$.

In order to show ${\mathsf {NP}}$-hardness of MaxEDP for ${r=1}$, we also reduce from Edge 3-Coloring in cubic graphs and perform a similar construction as described above: This time, we construct a bipartite graph *G* with one subset of the partition being ${\{v_1,v_2\}}$, the other being *V*(*H*), and the set ${\mathcal {M}}$ of terminal pairs being again specified by the edges of *H*; see Fig. 6a, c. This completes the reduction. The resulting graph *G* has feedback vertex set number ${r=1}$.

We claim that *H* is 3-colorable if and only if we can route ${n=|V(H)|}$ pairs in *G*.

In the forward direction, suppose that *H* is 3-edge-colorable by a proper coloring ${\varphi :E(H)\rightarrow \{1,2,3\}}$. For ${c \in \{1,2,3\}}$, let ${E_c\subseteq E(H)}$ be the set of edges that receive color *c* under ${\varphi }$. Then there is a routing in *G* that, for every ${c\in \{1,2\}}$, routes all terminal pairs ${\{(s,t)\in \mathcal {M}\mid \{s,t\}\in E_c\}}$ exclusively via the node ${v_c}$ (and thus via paths of length 2). Note that the terminals corresponding to edges receiving color 3 remain unrouted. The reasoning that the resulting routing is feasible is analogous to the case of ${r=2}$. To see that precisely *n* terminal pairs are routed overall, observe that, for each of the *n* terminals, exactly two of the three terminal pairs are routed.

In the backward direction, suppose that *n* terminal pairs in ${\mathcal {M}}$ can be routed in *G*. Since every terminal *v* in *G* has degree two, at most two paths can be routed for *v*. As *n* terminal pairs are realized, this also means that *exactly* two paths are routed for each terminal. Hence, none of the paths in the routing has length more than two. Otherwise, it would contain an internal node in *V*(*H*), which then could not be part of two other paths in the routing. Then this routing naturally corresponds to a partial edge-coloring of *H*, where any terminal pair ${\{s,t\}}$ routed via ${v_c\in \{v_1,v_2\}}$ implies that we color the edge ${\{s,t\}\in E(H)}$ with color *c*. Since each terminal *v* in *V*(*H*) is involved in exactly two paths in the routing, exactly one terminal pair for *v* remains unrouted. Hence, exactly one edge incident on *v* in *H* remains uncolored in the partial coloring. We color all uncolored edges in *H* by color 3 to obtain a proper 3-edge-coloring. $\square$

Thus, we almost close the complexity gap for EDP with respect to the size of a minimum feedback vertex set, only leaving the complexity of the case ${r = 1}$ open.

## Concluding remarks

In this paper, we examined the problems of routing terminal pairs by edge- and node-disjoint paths in graphs of bounded feedback vertex set number *r*. We observed that our obtained approximability bounds, expressed in terms of *r*, either strengthen best known bounds or they are almost tight. This leads us to the conclusion that the parameter *r* in fact captures the “difficulty” of disjoint paths problems.

In particular, for MaxEDP, we obtained a constant-factor approximation algorithm with congestion logarithmic in ${k+r}$, where *k* is the number of terminal pairs. This strengthens the bound obtained by directly applying the randomized rounding technique for LPs introduced by Raghavan and Thompson [40]. Though also we applied this technique, beforehand we appropriately modified the fractional LP solution by making use of the forest that one obtains when removing the feedback vertex set from the graph. For our next result, we used the solution above to extract ${{\text {OPT}}^{*}/\mathcal {O}({\sqrt{r}\log (kr)})}$ edge-disjoint paths out of it, where ${{\text {OPT}}^{*}}$ denotes the value of an optimum fractional solution. This strengthens, up to a logarithmic factor, the best known bound of ${{\text {OPT}}^{*}/\mathcal {O}(\sqrt{n})}$ [11]. We achieved our result by contracting “redundant” edges in the input graph and in the routing which lead to an “irreducible” routing from which we could greedily pick up our solution. The result shows that in order to improve the best known bound it suffices to focus only on graphs with feedback vertex set number close to *n*.

We also complemented the upper bounds with hardness results. We observed that the complexities of both problems, routing node-disjoint paths and edge-disjoint-paths, differ when *r* is constant. Whereas NDP [43] and MaxNDP are efficiently solvable for any constant *r*, EDP and MaxEDP are ${\mathsf {NP}}$-hard even for ${r=2}$ and ${r=1}$, respectively. Here, the complexity of EDP remains open for ${r=1}$ and we conjecture that this case can be solved in polynomial time. When considering *r* as part of the input, we can separate NDP and MaxNDP (if $\mathsf {FPT}\ne \mathsf {W}[1]$). We showed ${\mathsf {W}[1]}$-hardness of MaxNDP when parameterized by *r*, whereas NDP is fixed-parameterized tractable in *r* [43]. However, we were able to provide a fixed-parameter algorithm for the combined parameter ${k+r}$.
